# Computational workflow for the fine-grained analysis of metagenomic samples

**DOI:** 10.1186/s12864-016-3063-x

**Published:** 2016-10-25

**Authors:** Esteban Pérez-Wohlfeil, Jose A. Arjona-Medina, Oscar Torreno, Eugenia Ulzurrun, Oswaldo Trelles

**Affiliations:** 1 Department of Computer Architecture, University of Málaga, Boulevard Louis Pasteur 35, Málaga, Spain; 2 Advanced Computing Technologies Unit, RISC Software GmbH, Softwarepark 35, Hagenberg, Austria

**Keywords:** Metagenome analysis, Differential abundance, Annotational mapping, Mapping over specific regions, Open platform

## Abstract

**Background:**

The field of metagenomics, defined as the direct genetic analysis of uncultured samples of genomes contained within an environmental sample, is gaining increasing popularity. The aim of studies of metagenomics is to determine the species present in an environmental community and identify changes in the abundance of species under different conditions. Current metagenomic analysis software faces bottlenecks due to the high computational load required to analyze complex samples.

**Results:**

A computational open-source workflow has been developed for the detailed analysis of metagenomes. This workflow provides new tools and datafile specifications that facilitate the identification of differences in abundance of reads assigned to taxa (mapping), enables the detection of reads of low-abundance bacteria (producing evidence of their presence), provides new concepts for filtering spurious matches, etc. Innovative visualization ideas for improved display of metagenomic diversity are also proposed to better understand how reads are mapped to taxa. Illustrative examples are provided based on the study of two collections of metagenomes from faecal microbial communities of adult female monozygotic and dizygotic twin pairs concordant for leanness or obesity and their mothers.

**Conclusions:**

The proposed workflow provides an open environment that offers the opportunity to perform the mapping process using different reference databases. Additionally, this workflow shows the specifications of the mapping process and datafile formats to facilitate the development of new plugins for further post-processing. This open and extensible platform has been designed with the aim of enabling in-depth analysis of metagenomic samples and better understanding of the underlying biological processes.

**Electronic supplementary material:**

The online version of this article (doi:10.1186/s12864-016-3063-x) contains supplementary material, which is available to authorized users.

## Background

The purpose of metagenomics is to identify the species present in an environment. Different types of studies can be performed based on metagenomics. Some examples include the analysis of changes in the presence of species in a given environmental sample and the use of phylogenetic analysis to follow up the spread or determine the origin of a species. A large number of tools are emerging in the form of stand-alone programs (e.g. MEGAN [1]), interoperable Web services (e.g. MG-RAST [2]) or tools accessible through the Internet (e.g. EBI Metagenomics [3]).

MEGAN performs taxonomic analyses of a metagenome by mapping reads to different taxa based on BLAST [4] search results and the NCBI taxonomy. To perform this task, the program runs the lowest common ancestor (LCA) algorithm to classify input reads. Most metagenomic tools are constructed following a workflow scheme offering distinct stages of data processing. In this line, the open-source EBI Metagenomic workflow is split into two branches following the quality control step. The first branch performs taxonomic classification based on 16S rRNA, whereas the second branch performs functional analysis based on protein-coding sequences. Unannotated reads are kept out of the pipeline. However, these reads should be taken into account for whole metagenomic analysis in order to improve the accuracy of taxonomic classification and better understand the roles of species in environmental samples.

The number of comparative metagenomic tools is the key point of the metagenomic RAST (MG-RAST) platform. MG-RAST builds clusters of proteins at a given percentage of identity level using QIIME [5]. Once built, the longest sequence of each cluster is subject to similarity using sBLAT, an implementation of the BLAT [6] algorithm. MG-RAST also uses the NCBI taxonomy for taxonomic classification. Functional profiles are available through comparison against data sources that provide hierarchical information. Abundance profiles are the main output for displaying information on datasets. The MG-RAST annotation pipeline does not generally provide a single annotation for each submitted fragment of DNA. Steps in the pipeline map a read to multiple annotations and vice versa. Data privacy is one of the concerns of the scientists using this tool. Firstly, they are reluctant to upload their unpublished and/or confidential data to a public website. Secondly, the priority of analysis requests to the website is subject to the level of confidentiality of input data (with lower priority and therefore longer waiting times for private data).

Recently, a new DNA sequence analysis workflow called META-pipe [7] has been developed to find novel commercially exploitable enzymes from marine microbial communities. META-pipe uses tools such as MetaGeneAnnotator (MGA) [8] and Protein BLAST to identify sequences found in the UniProtKB database. MGA is a new version of MetaGene [9] where a prophage gene model is offered in addition to bacterial and archaeal models. MGA uses di-codon frequencies estimated by the GC content of an input sequence to map genes using regression models. In addition, MGA offers an approach for the analysis of ribosomal binding sites (RBSs) to detect specific patterns of ribosomal sequences in species. However, due to their tendency to undergo highly degenerative changes, RBSs are particularly difficult to identify [10]. In the line of pipelines used to facilitate the comparative analysis of high throughput sequencing, MOCAT [11] is a modular tool for processing raw sequence reads produced by the Illumina technology [12]. The main steps in MOCAT are 1) read trimming and filtering 2) read assembly, 3) gene prediction and 4) estimation of taxonomic abundance profiles.

The fine-grained metagenomic analysis workflow developed by our group can operate over a user-defined collection of genomes -thus accelerating the computational process- and with the advantages of being able to map reads over unannotated regions of genomes. Our software provides different mapping methods and different mapping alternatives apart from the best read-genome matching. In addition, it provides information about the quality of mapping and about differences between mapping options. Unlike the methods currently available, which deduce that a species is not present in a sample when its abundance is low (in number of reads), the proposed method can detect low-abundance species by finding reads mapping to particular specific regions of genomes. In addition, the developed workflow is an open platform composed of an expandable set of separate modules that use well-defined format datafiles. This enables the easy on-demand incorporation of new processing tools. Along with low-abundance species support, other tools have been included to verify the correctness of taxonomic assignation and extrapolate DNA sequencing data to gene expression levels.

## System and methods

### System and requirements

**Fig. 1:**
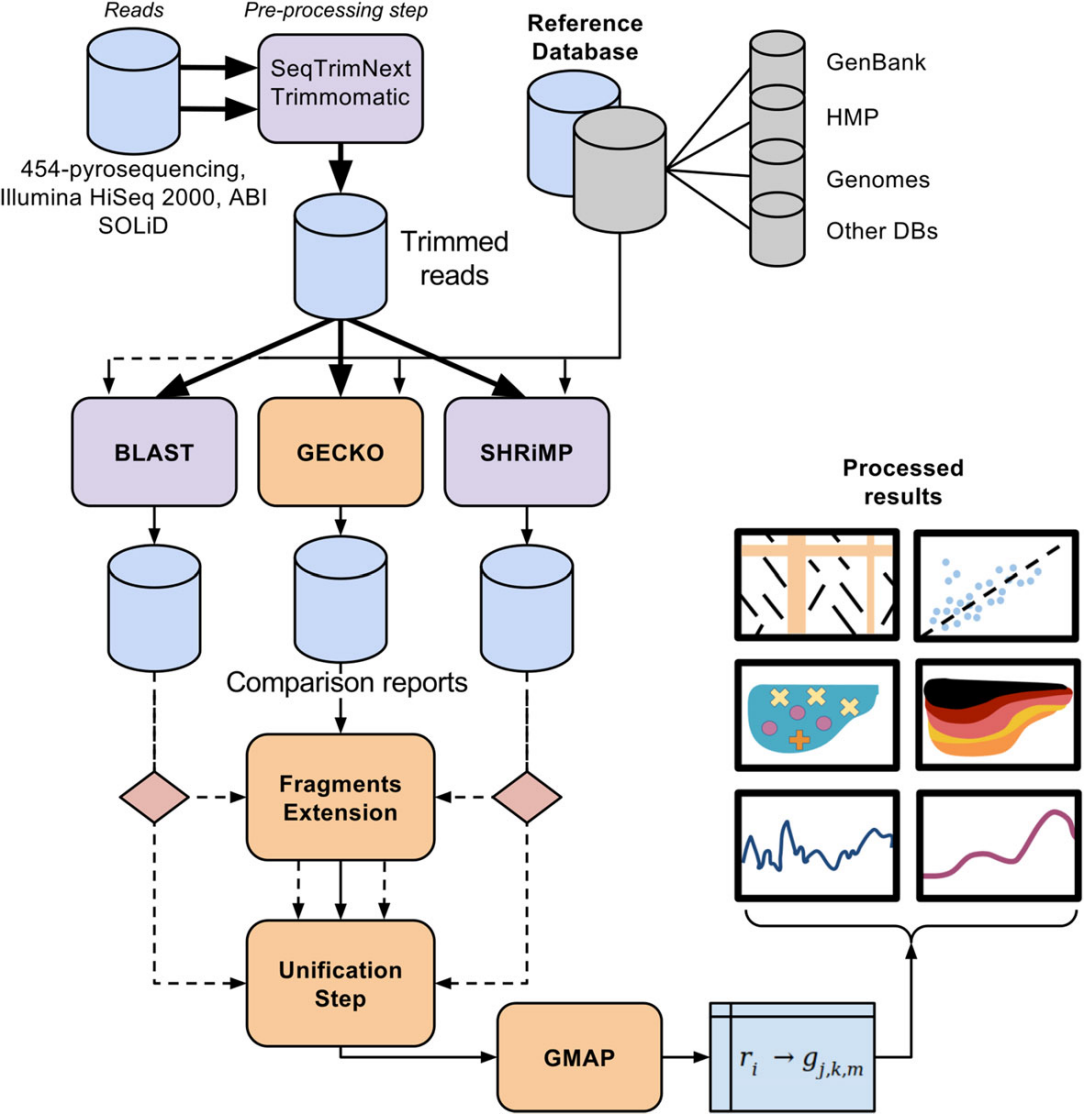
The Workflow diagram. *Top*: Quality control layer and input files. *Center*: Comparison software layer. *Bottom*: Mapping kernel (GMAP), which provides open-source datafile definitions and enables many on-demand post-processing experiments (*Right*)

The designed workflow (see Fig. 1) includes all the needed steps for data processing. The quality control step can be performed using SeqTrimNext [13] for the case of 454-pyrosequencing [14] reads, whereas Trimmomatic [15] can be used for Illumina reads. These programs are available in our workflow implementation under our Galaxy [16] instance. With regards to the sequence comparison algorithm, we suggest to use the GECKO [17] package to accelerate the process. Since several metagenomic packages for 454-pyrosequencing reads are based on the matches provided by a BLAST run, the developed workflow offers a parser to translate BLAST’s output, and therefore the same strategy (parsing) can be used when other sequence comparison software is employed. In addition, the sequence comparison tool SHRiMP [18] is included along with a parser that is also available and described in the Additional file 1.

In the line to offer a broad scope of the presented software, the proposed workflow can handle sequences of different length obtained with different sequencing technologies (e.g. SOLiD [19], Illumina, 454-Pyrosequencing). For instance, in the case of colour-space reads these can be compared using SHRiMP, which natively supports colour-space reads. The proposed method focuses in the comparison and mapping procedures, while the pre-processing steps can be carried out with common publicly available software.

The workflow operates over a user-defined collection of genomes. This database might as well be a custom selection of genomes which hold particular interest, a pre-selection of the most common species for the type of metagenome analysis, or even a complete database such as GenBank [20].

The workflow is specialized in matching (reads-species mapping) and post-processing procedures, which require the following input: (1) Sequence comparison files, (2) taxonomic description of the reference dataset, and (3) annotation files for the genomes (optional, only needed for post-processing).

(1) Sequence comparison files: the workflow has been designed so that it is compatible with any sequence comparison software (i.e. BLAST family, FASTA family, proprietary software, etc). The default comparison software used in this workflows is GECKO, however, the user can employ other packages. To include other comparison software a format conversion program would be needed. A parsing conversion system for BLAST is already included in the workflow. The parsing module converts sequence comparison files to a format composed of headers (read-genome tuples) followed by rows, where each row represents a fragment for the tuple. Fragments belonging to a read-genome match are defined by a 12-tuple:

$${} \begin{aligned} t_{n,k}^{12} =& (k, score, identities, length, similarity, igaps, egaps,\\ &strand, rStart, rEnd, gStart, gEnd) \end{aligned} $$

Where *k* is the *k*−*t* *h* fragment reported by the read *n*(see Additional file 1 for further reading). The fields *r* *S* *t* *a* *r* *t*,*r* *E* *n* *d*,*g* *S* *t* *a* *r* *t*,*g* *E* *n* *d* represent the anchoring positions in the read *r* and genome *g*. Reversed fragments are found by comparing the read with the reverse complement of genome *g*. Notice that *rEnd* and *gEnd* are redundant for ungapped fragments, but necessary for gapped fragments. (2) A taxonomic description file allows the customization of hierarchical relationships between organisms in the reference database as assigning strain relationships between species or separating strains that belong to a common ancestor. Such file can be generated automatically using a module of the workflow and/or can be manually built to insert customized relationships between species. The format of the file generated is a text file including a 5-tuple per line, each tuple being a new genome:

$${} \begin{aligned} t_{n,m}^{5}=& (n, m, genome\ accession\ number, genome\ name,\\ &length) \end{aligned} $$

Where *n* and *m* are the specie and subspecie id’s. These can be used to set up custom boundaries. For further details, please see “Taxonomy files” in the Additional file 1).

(3) Annotation files are used to carry out all coding region-related computations in the post-processing phase. Therefore, these files are optional and should be included if annotation post-processing modules are to be used. As in the case of comparison software, a parsing system is implemented; e.g. a parsing system for GenBank’s annotation files has been included in our workflow.

### Extension of ungapped HSPs

Comparative analysis of metagenomes is an expensive computational process that involves comparing a large set of DNA fragments against an enormous database of candidate sequences (genes, proteins or genomes). It should be noticed that, by definition, bacteria in the metagenome are uncultured species and the sequences in the databases that already exist are not –most likely– the corresponding to the species in the metagenome. Even some large mutations (inversions, deletions, etc) can happen regularly. Therefore a more flexible matching is proposed, which differs from an assembly by mapping in which there are quite close representatives of the sequenced bacteria.

Thereby we included the option of using a custom glocal [21] alignment, which yields longer fragments and larger evolutionary gaps. This method generally improves mapping results, as global alignment methods are less accurate when identifying species.

Once local alignments are calculated (using GECKO, BLAST or any other similar program), fragments are extended by joining those that are close enough according to a given maximum-gap parameter. This is done by calculating the Needleman-Wunsch matrix between the start and the end of the matching read within the genome region with a customized implementation.

Furthermore, glocal alignment can be performed by combining the local alignments produced by alignment tools such as BLAST or GECKO with the provided custom glocal alignment. All parameters can be user-defined, thus providing data processing flexibility. Table 1 shows an example of candidate fragments that are extended to conform a glocal alignment.

**Table 1:** Before-and-after extension example of a read with two candidate fragments to be joined

Before extension of local or ungapped alignments											
029701.102903 — NC_004663.1 — Bacteroides thetaiotaomicron 897405											
N	SCORE	IDEN	LEN	SIM	IGAPS	EGAPS	STRA	R1	R2	G1	G2
1	-	134	145	84	0	0	Plus ∖Plus	1	133	1631420	1631563
2	-	94	104	80	0	0	Plus ∖Plus	147	249	1631564	1631666
After extension (“glocal-like” alignment)											
029701.102903 — NC_004663.1 — Bacteroides thetaiotaomicron 897405											
1	-	224	248	90	1	2	Plus ∖Plus	2	249	1631420	1631665

In the top, the table before extension. Fragments (1) and (2) are separated by a relatively small gap of 14 base pairs (The ending read coordinate R2 of (1) is 14 base pairs away from R1 in (2)). These fragments represent an example of candidate fragments. The after subtable (bottom) displays the resulting extended fragment and shows a longer alignment with still high similarity and a low number of gaps (one opening gap and two extension gaps). The score is calculated afterwards (See “New score and expected value calculation”).

### New score and expected value calculation

The extension of fragments requires the re-calculation of fragment scores to identify the best match out of a list of candidates during the mapping process. The properties of the extended fragment, namely length –of bases–, number of identities and inserted gaps stand for the raw score. The raw score has to be normalized in order to obtain the expected value of a reported fragment. This is performed using K and Lambda parameters using a similar approach to that of the BLAST family. K and Lambda parameter are calculated as described in [22].

To compute the raw score of the extended fragment produced by our custom glocal alignment we apply a traditional affine scoring model (with open and extension gap penalties), as shown in the following formula:

1 $$ \begin{aligned} RS &= I * M_{r}+ (L-(G_{i}+G_{e})-I)\\ &\quad* M_{p} + G_{i} * P_{i} + G_{e} * P_{e} \end{aligned}  $$

Where *RS* stands for “Raw Score”, *I* for the total number of identities in the fragment, *M*_*r*_ for the match score, *L* for the total length of the fragment in base pairs, *G*_*i*_ for the total number of open gaps in the fragment, *G*_*e*_ for the total number of extension gaps in the fragment, *M*_*p*_ for the mismatch penalty, *P*_*i*_ for the penalty of an open gap and *P*_*e*_ for the penalty of an extension gap.

### Mapping

The mapping module (GMAP) process offers a three-level mapping option that not only discovers highly abundant species that hide others in terms of abundance due to high similarity or uncertainty in the alignment, but to also obtain quality distance measurements between the best 3 candidates for every match. The top three candidates are selected based on identity and coverage thresholds and expected values. Moreover, users can perform different mappings by restraining subsets of reads using different thresholds.

In a scenario with a highly abundant organism, further analysis can be performed by only considering certain genomes using certain options e.g. to observe differences and extract statistical indicators of close candidates.

### About the mapping decision

Every read yields a list of reported fragments to which the following algorithm is applied.

1. Filtering step: A filtering step allows the researcher to consider only a subset of reported fragments, enabling a levelling up mapping method. If a fragment does not reach pre-filtering thresholds, it will be discarded. Such filtering allows a two phases pre-filtering:
  - Coverage threshold phase: The length of the match divided by the length of the read.
  - Identity threshold phase: The number of identities in the match divided by the length of the match.
2. Repeat this step for 3-option mapping and, if fragments are still active: Select the fragment with the smallest expected value and if it is lower than the maximum allowed expected value. This fragment is included in the mapping file as first, second or third candidate depending on the number of options chosen and the genome is inactivated for the next option iteration.
3. If no more fragments are still active or none of them exceeds the thresholds, the read is decided with either no mapping option or up to 3 mapping options.

See “Mapping decision and fragments” in the Additional file 1 for more information.

## Results and discussion

Rather than developing a monolithic application with graphical interfaces, we opted for a simple pipelining procedure in which new software modules can be used to exploit results. To facilitate user interaction, a complete web-based interface has been developed based on Galaxy workflow manager, which enables users to easily run their analyses in both a local instance or in dedicated servers. In addition, a User Guide [23] is available. Regarding software modules, all specifications about input and result file formats are shown, facilitating the use of third-party software, such as common graphical libraries and spreadsheets.

The results given by our workflow software are illustrated by an experiment where two collections of 6 metagenome samples each were extracted from faecal microbial communities of adult female monozygotic and dizygotic twin pairs concordant for leanness or obesity and their mothers [24]. Raw data (i.e..sff files) were obtained by 454-pyrosequencing, and inherent artefacts or low-quality sequences were further filtered and removed using Replicates [25] software and SeqTrimNext (See “Filtering and trimming parameters” in the Additional file 1 for used parameters). The average size of the read collection ranged from 172 bp to 237 bp after quality control and sequence trimming. The total number of sequences was 2,724,867 for lean metagenomes and 2,972,697 for obese ones. For testing purposes, in this technically-oriented paper we opted to design a synthetic case-control study of two metagenomes by joining samples from lean and obese individuals.

### Reads-abundance and taxonomy classification of reads

**Fig. 2:**
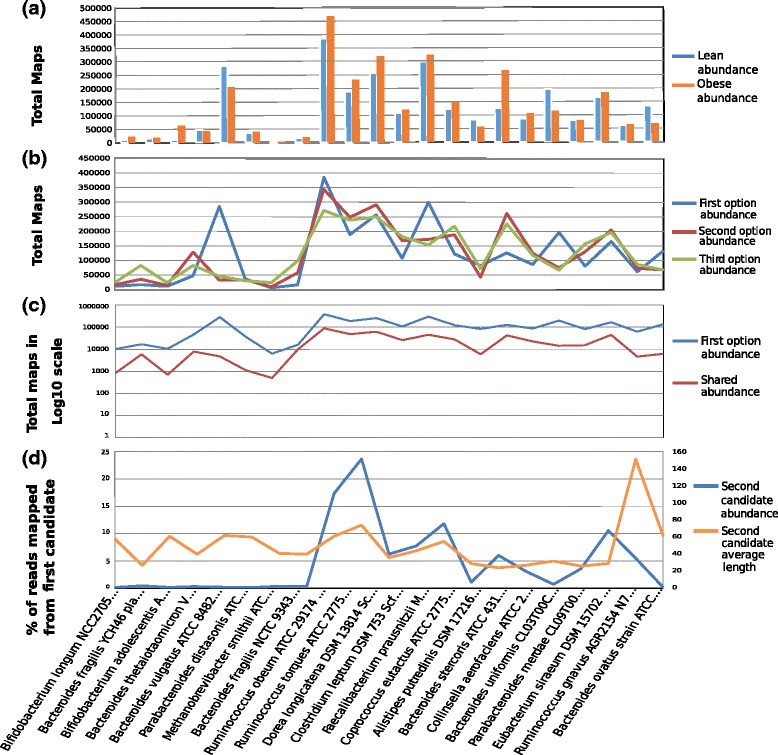
Three-options mapping analysis. Some data from GMAP-based mapping analysis. **a** Abundance plot for the averaged Lean (*blue*) and Obese (*orange*) metagenomes of the most read-abundance genomes. The plot depicts total mapped reads per specie in the two averaged metagenomes. **b** Three-option abundance by organism. In blue, total first option abundance, (number of reads assigned). In red and green, the number of times an organism was the second and third best candidate for a read. Bacteria with red or green peaks reveal that another organism is probably hiding them (regarding abundance) and there is not a direct consensus. **c** Total reads assigned in log10 scale per species as best candidate (*first option, blue*) and from that total, the number of reads that had two very similar candidates (defined as a distance in terms of identity, length and coverage) from the second best candidate (*in red*). **d** An exhaustive-one-vs-all user-defined analysis where a bacterium is compared against all species in the database. The peak in the plot (near the middle) is the analyzed genome, Ruminococcus obeum ATCC 29,174. This particular scenario depicts a comparison of the target genome against all species by length and abundance. In blue, the percentage of reads that were mapped as second candidate when the best candidate was the target genome. In *orange*, the average length of such mapped reads

The analysis of the species present in metagenomic samples enables taxonomic classification based on abundance of mapped reads. Information about the species present in metagenomes and variations across a collection of species is yielded by GMAP in Comma Separated Value (csv) format files that can be edited using common spreadsheet software (see Fig. 2 a).

Abundance data are primarily used to determine the species that are present in a metagenomic sample and can be exploited in comparative studies on the over- or under-abundance of species in different samples. However, abundance data does not provide information on the quality and certainty of mapping. This lack of reliability can be partially compensated by using the n-mapping method.

**Three-options mapping analysis** Our software has the ability to perform the mapping of reads through a multiple-level strategy. After the best read-genome mapping value is used, the used fragment is inactivated and the genomes belonging to different strains of the same species are optionally inactivated, and the process is repeated. This way, we get the second, third and subsequent best read-genome mapping values. A long separation between the mapping options provides stronger evidence supporting the validation of the mapping procedure.

When comparing 3-mapping options, the detection of peaks in second or third options means that a particular species is repeatedly the second or third candidate (see Fig. 2 b). These peaks suggest that strong similarities exist between a specific pair of species and careful examination is required since the accuracy of mapping is not certain. For instance, it would be interesting to study if the alphabetical order of the BLAST output for sequences with the same expected value is affecting the mapping. These observations can be supported by the analysis of mapping precision (see Fig. 2 c), which considers the closest reads given a distance parameter and shows the separation in mapping length, the number of identities, or any other chosen parameter between the assigned read and its second best candidate. Additionally, this separation shows the extent of differences between first and second candidates, and therefore is another indicator of the quality of mapping.

In addition, the 3-mapping approach allows to assess the mapping certainty at both reads and species level; at read level by comparing fragments quality indicators of particular genomes against the rest (see Fig. 2 d), and at species level by comparing the abundance levels of the different options for the particular genomes. For example, for all reads mapped over a given genome, information about the identity and coverage level of the second and third mapping option would provide information about the quality or certainty of the first option.

On the other hand, in a joint analysis of the Fig. 2 b and c, no peaks in second or third options, along with a larger separation gap on mapping precision analysis suggest that the accuracy of mapping is high, which reduces the random assignment of reads to genomes and, therefore, the results obtained are more reliable.

Figure 2 c displays the number of reads assigned to each species and, in relation to each assignation, the times the second option was almost as good as the first (namely, “shared” reads). The fact that the blue and red lines of two species are close to each other suggests that mapping is not accurate and careful examination is required.

**Fine-grained tuning and closer examination** In a scenario where a specific species has been the second option a higher number of times compared to the first option, as discussed in the previous Section, the mapping should be exhaustively analyzed and compared with other species. Such analysis would provide more certainty of the presence of a low-abundance genome by checking the properties of its matches, and would enable contrasting the variances in the matches between a high-abundance genome and its best second option. Moreover, it is possible to perform a one vs. one, one vs. some, or, some vs. some comparative analysis of the target species. This type of analysis can be performed based on any of the properties of the mapped reads, such as length, similarity, coverage, or any user-defined properties. This information is particularly useful when the first and second mapping options identify different species (in some cases, remotely-related species).

Figure 2 d illustrates how a number of reads map to very similar sequence regions shared by different species (due to high similarity at genome level –i.e. conserved genes). For example, the mentioned Figure displays the second mapping option of the reads that were mapped as first solution to the Ruminococcus obeum ATCC 29,174. The blue peak in the middle of the plot stands for almost 25 % of the reads assigned to Ruminococcus as first option and to Dorea longicatena DSM 13,814 as second option, which evidences strong similarities in several areas of the two sequences. Additionally, the orange peak at the right side suggests a longer alignment in the second option –Ruminococcus gnavus AGR2154–, thus requiring in-depth analysis of such reads.

**Statistical significance of variations between samples** The presented software can provide statistical data on a number of aspects or characteristics, such as the Z-score test to detect significant variations in the abundance of species in different experimental conditions; or to contrast the significance of the variation at a species level between samples calculating the *p*-values. An interesting example is case-control studies in which differences in reads abundance along genomes can be identified. Z-scores provide accurate information on the significance of such differences (see “Statistical Significance” in the Additional file 1 for more information).

### Genome-specific experiments and quality assessment

**Reads mapping to specific regions of genomes** Besides the proximity measures provided by three-option mapping, there is another important aspect concerning the provision of evidence about the presence of species with low-abundance of reads in the metagenome. The main idea is to find regions in a particular organism that do not exist or do not share similarity at all with other organisms present in the collection of genomes. To accomplish this, *N*−1 comparisons between the reference genome and the *N* genomes contained in the collection are performed using GECKO. This process yields the detected regions and the assigned reads that have been mapped to these regions.

The extracted reads mapped to these regions provide strong evidence on the presence of low-abundance species in the metagenomic sample, since the mapped read does not fit over other genomes (see “Reads mapping to specific regions of genomes” in Additional file 1 for more information).

**Fig. 3:**
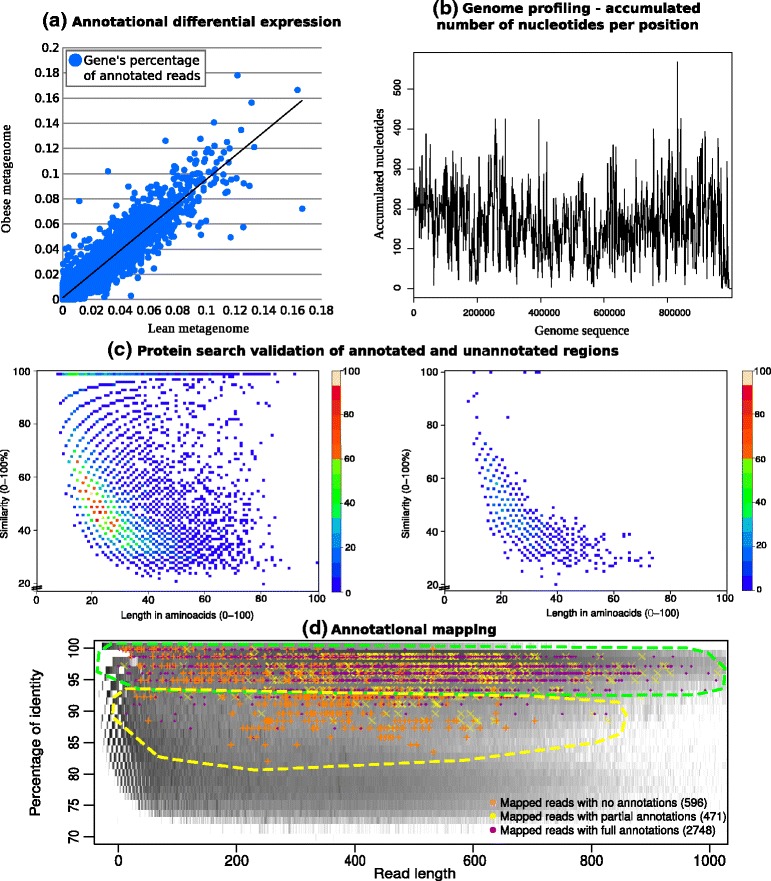
Genome-specific experiments. Some of the results oriented at a genome-specific-level. **a** DNA-seq differential expression plot. Each point represents an annotated region for a particular genome. In the x-axis and y-axis, the percentage of reads that are mapped to each annotated region divided by the total mapped reads. **b** Accumulated reads mapped onto each position of the genome smoothed using a window of size 10000. In the x-axis, the genome bases from 1 to a portion of its length. In the y-axis, absolute accumulated number of reads mapped. **c** This plot shows how proteins found by searching with annotated (*Left*) and non annotated (*Right*) reads accumulate along similarity and length. The annotated search depicts higher length and similarity matches, resembling Sanders curve (reference in the main text), whereas non-annotated search shows mostly non significant matches. **d** Annotation mapping. This plot shows reads mapped to a particular genome distributed by annotation properties. The three groups are plotted in different colours and shapes, namely *a* orange crosses for unannotated reads, *b* yellow crosses for semi-annotated reads and *c* purple points for fully-annotated reads. The background grey area represents the accumulation of reads for the whole mapped metagenome in logarithmic scale; thus, darker areas represent higher accumulation

**Differential abundance in annotated regions of genomes** Another useful tool is the comparison plot of abundance of annotated regions (potential coding regions that could change abundance values in different samples). This assay is conducted on a particular genome by only considering the reads mapped to annotated regions of the genome and comparing abundance between different samples in the same way as RNA-seq transcriptome expression analysis is performed. Differences in the abundance of reads mapped to annotated regions –when sampling genomic DNA– might be related to environmental changes. This hypothesis is based on the experimental resemblance of the differential expression plot of annotated regions when two samples whose environmental conditions change are compared. Figure 3 a suggests that some annotated regions are being over- or under-represented, thus suggesting that abundance in annotated regions may be related to variations in the samples.

**Genome profiling of mapped reads** A genomic profile of mapped reads is the accumulated number of reads mapping to a given position within the genome. Accumulated histograms of abundance of reads provide information about the number of reads at region level, and therefore about variations in such accumulation (in case-control experiments). In principle, when working with genome sequencing, a more or less flat profile would be expected, as opposed to transcriptomics sequence data. The genomic profile helps detect highly active regions or different number of copies in such regions. This visualization tool (see Fig. 3 b) shows how reads are distributed in a particular species and whether the assigned reads are present along the whole genome or only in the most active areas. Another possibility offered by this tool is that it helps the user decide whether to perform or not a pre-assembly of the reads mapped to a specific genome to support the connections found between reads.

**Extensive and further verification** We propose that the distribution of fragments based on the comparison of reads versus genomes is now divided into two different distributions, as seen in Fig. 3 c. Additional verification was performed by representing the matching values for reads falling into annotated and unannotated regions. This is obtained by blasting the set of annotated and unannotated reads mapped to a given genome against a database of proteins –such as swissprot [26]. As expected, different distributions are obtained, which evidences the suitability of using different thresholds. This affirmation is supported by the different levels of sequence conservation in annotated and unannotated regions.

**Mapping over annotated regions of genomes** Annotation mapping is another example of in-depth analysis of a specific genome and, in particular, of low abundance ones. Our workflow uses all the reads assigned to a genome and divides them into three groups: (1) No annotations, in the sense that the annotation files obtained did not contain any annotation at the position where the read was mapped; (2) Semi-annotations, when a part of the mapped read contains annotations, and (3) Full annotations, when the whole read contains an annotation.

These three groups are plotted onto the whole mapped metagenome distribution (see Fig. 3 d). The background grey area represents the accumulation of reads for the whole mapped metagenome in logarithmic scale; darker areas represent higher accumulations. The identity-length distribution of reads for all fragments (with any filtering) is provided by GECKO and can be partially obtained from data evidencing significant alignments yielded by other programs (BLAST) (which can be tuned to also report random distribution). The rationale of this result comes from the experiments of Sanders et al. [27] and Rost [28] that significance is related to the tail of the distributions. Therefore, displaying mapping values on the grey area distribution provides first-glance information about the accuracy of mapping.

### Comparison with other metagenomic tools

In order to prove that the results of the proposed workflow are consistent with those of other metagenomic analysis software suites (in terms of abundance in the taxonomic classification), the following test was performed using results from BLASTn based on metagenomic samples from faecal microbial communities. Both, our workflow (MG workflow) and MEGAN were executed using the same input from BLASTn and ran with default parameters (available in the Additional file 1 under “Comparison with MEGAN”).

**Fig. 4:**
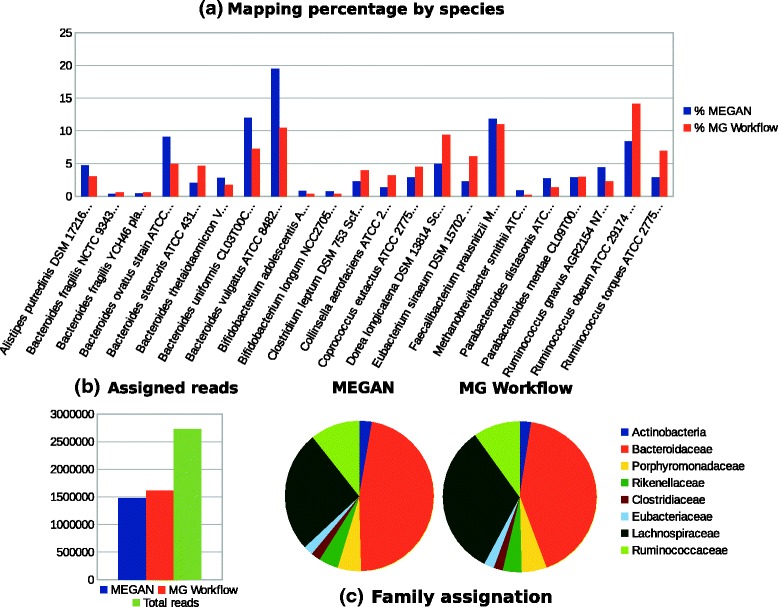
MEGAN and MG Workflow comparison. Comparative analysis for the lean metagenome shows similar mapping abundances. **a** Abundance plot by species in percentages. **b** Total reads assigned by each method and total number of reads in the metagenome. **c** Abundance chart by family (except Actinobacteria, shown as Phylum)

On comparison of the lean metagenome based on MEGAN, the abundance plot (see Fig. 4 a) shows similar results to ours. Standard deviation from ratios (using abundance data provided by MEGAN and by our workflow) was 0.25, which is not significant enough to identify relevant variations (see Fig. 4 b, c). However, whereas the analysis of a metagenome using MEGAN can last nearly an hour, our MG workflow took about six minutes to analyze the obese metagenome and five minutes for the lean one when the comparison had been done with BLAST. With GECKO, the duration of the process was further reduced, taking about only one minute for the lean sample and three minutes and a half for the obese metagenome. Runtime executions were measured using a regular Intel i5 machine with 4 GB of RAM.

## Conclusions

Metagenomics is an effervescent field and there are still a number of questions that need to be addressed before a stable version of data analysis software becomes available. Currently, metagenomic analysis tools generally represent a closed environment and offer few configuration options and limited extension possibilities. Our aim was to develop a software framework to which other modules could be added. An additional motivation to develop this software was the need for software sensitive enough to detect the presence of low-abundance species. Finally, our intent was to provide data in standard and editable formats that facilitate further analysis with external software.

The proposed workflow software offers several notable advantages over the software currently available in the market. Firstly, the use of GECKO enables this software to compute similarity searches in the samples against a collection of genomes in a reasonable time. We found that better results are obtained if a collection of genomes –rather than genes or proteins– is used. At least this was the case when not all genes/proteins from the genomes were registered in reference databases. Moreover, if genomic samples are used (not only transcriptomics), a significant amount of reads would map to unannotated regions, and therefore they would not match to databases composed of genes or proteins.

Providing different mapping alternatives helps set up a sort of quality measures of the mapping process based on abundance differences across mapping alternatives. In addition, the study of the different alternatives could reveal hidden interactions or shared similarities between species that cooperate in some aspects.

The proposed software is designed to provide evidence of the presence of low-abundance species by finding particular specific regions of genomes with mapped reads. These mapped reads provide strong evidence of the species present in samples. The methods developed for assessing and evaluating the quality of mapping also improve accuracy and reliability in terms of the identification of the species present in a sample.

From our perspective, the most important contribution of this workflow software is that it offers the possibility of incorporating new software to extend the analysis workflow by showing datafile specifications enabling fine-grained metagenomic data analysis.

## Additional file

Additional file 1 Supplementary material. (PDF 1269 kb)
