# Supplementary material for: Computational workflow for the fine-grained analysis of metagenomic samples
Source: BMC Genomics. 2016 Oct 25;17(Suppl 8):802. doi: 10.1186/s12864-016-3063-x (PMC5088524; doi:10.1186/s12864-016-3063-x)
Supplement: Supplementary file 1 — Supplementary material. (PDF 1269 kb) [file 12864_2016_3063_MOESM1_ESM.pdf]

Computational workflow for the fine-grained analysis of metagenomic samples

Esteban Pérez-Wohlfeil[1], José Arjona-Medina[2], Óscar Torreño-Tirado[1)], Eugenia Ulzurrun[1], Oswaldo Trelles[1]

1. Department of Computer Architecture, University of Malaga; Malaga, Spain
2. Advanced Computing Technologies Unit, RISC Software GmbH; Hagenberg, Austria

Supplementary Material

- Why is it fine-grained?
- Additional files
  - Pre-processing step
  - Filtering and trimming parameters
  - Base software in metagenomic analysis packages used in sequence comparisons
  - MOCAT usage in the taxonomical estimation of the abundance of reads
  - Reference database
  - Contrasting signals between a highly abundant taxon and a closely related one
  - Handling of blocking organisms
  - Statistical significance
  - Reads mapping to specific regions of genomes
  - Visual exploration of metagenome distributions
  - Coverage curves
  - Taxonomy files
  - About the parsing format in the workflow
  - Details of the extension of ungapped HSPs
    - Working example
  - Calculating the score and expected values after the extension
  - Mapping decision and fragments
  - Comparison with MEGAN 5
- Core-datafile definitions
  - Mapping structure
  - Gene structure
  - Genome-representation structure
- Post-processed-datafile definitions
  - Mapping results and the abundance file
  - Genbank parsed datafiles
  - Genome accumulated profiles
  - Matrix files
- Workflow extension and re-implementation example
  - Download and gather the code

[Creating a new module]
[Simple template]
[Adding some basic code]
[Compiling and executing]
[Appendix 1 - Reference database used in the experiment]
[References in Table 2]

Why is it fine-grained?

Although the etymology comes from sandpaper to refer rough (coarse-grained) or smooth (fine-grained) paper; nowadays the term is used in different contexts. When used in the context of "fine-grained control", for example, it carries the connotation of "very precise": a volume knob that gives you fine-grained control means that you can set your volume to the exact level that you desire, you don't have to choose only between "too quiet" and "too loud"; and in computer sciences is used for instance in the tasks distribution, that can be “coarse” or fine-grained. In the last case it represents an increase in the tasks-scheduling cost, but benefit of getting better control. This metaphor extends into other tools where the tools offer very fine control details in order to achieve specific results.
Being more specific, by “fine-grained” we refer to the optional post-processing steps aimed at finding evidence to support low-abundant genomes, since most metagenomic analysis tools treat these as statistically insignificant.

We have used the expression “fine-grained” in the sense that the system already contains a set of tools for very detailed data analysis, but even more, it can incorporate as many tools as needed in order to enhance data analysis.

Additional files

Pre-processing step

Although the pre-processing itself is not part of the workflow, we have made available quality control tools in the workflow to perform the trimming and filtering steps on 454-pyrosequencing and Illumina reads. SeqTrimNext has been included to pre-process the 454 reads whereas Trimmomatic has been included to pre-process Illumina reads. Please see the Guided Galaxy Exercise[1] and/or the Command-line Guided Exercise[2].

---

[1] http://bitlab-es.com/gecko/documents/Galaxy%20Guided%20Exercise.pdf
[2] http://bitlab-es.com/gecko/documents/METAGECKO-GuidedExercise.pdf

Filtering and trimming parameters

[Quoted from paper] Raw data (i.e. .sff files) were obtained by 454-sequencing, and inherent artefacts or low-quality sequences were further filtered and removed using Replicates software and SeqTrimNext [Quoted from paper]

Parameters for Replicates software:

Parameter	Value
Sequence identity cutoff	0.9
Length difference requirement	0
Initial base pair requirement	3

Table 1: Parameters used for Replicates software

Parameters for SeqTrimNext software:
The template *"genomic_short_reads.txt"* available at http://www.scbi.uma.es/ingebiol/commands/seqtrimnext/jobs/new was used to perform the trimming. All other parameters were left to default.

Base software in metagenomic analysis packages used in sequence comparisons

Table 2 shows the most widely-used metagenomic analysis pipelines and/or packages, the type of analysis that they perform along with the core comparison tool used for comparing genes, proteins, etc., the reference database used and the main steps where these comparisons take place.

Metagenomic Analysis Pipeline	Type of analysis	Core comparison tool used	Database	Step where it is used
MG-RAST [1]	(4)(5)	BLASTX	SEED	Screening potential proteins encoding genes.
MEGAN 1-4 [2]	(3)(4)	BLASTX	Any	Homology search.
MEGAN 5 [3]	(3)(4)	BLASTX, DIAMOND	Any	Homology search.

EBI Metagenomics [4]	(2)(5)	rRNAselector and InterProScan 5	Subset of InterPro databases	Ribosomal RNA prediciton and function prediction.
MOCAT [5]	(1)(6)	SOAPAligner2	Consortium Human Reference, Any	Remove reads that map to the human genome and reads abundance estimation.
Parallel-META [6]	(1)(2)(5)	MEGABLAST	Greengenes	Mapping 16S rRNA fragments to Greengenes.
META-PIPE [7]	(1)(5)	MetaGene, rRNA Selector, BLAST, Priam, Interpro	UniprotKB	Gene prediction and identification of enzymes for functional analysis.
CARMA 3 [8]	(3)	BLAST, HMMER3	Any	Homology search.
Phymm [9]	(3)	BLAST, IMMs	NCBI RefSeq	Homology search.
MetaMine [10]	(1)	BLAST	Microbial Ecological Genomics DataBase	Gene pattern search.

Table 2: Distinct metagenomic analysis packages, their area of application, algorithms and databases used. Legend of the area of application: (1) Gene calling, (2) Microbial diversity, (3) Sequence similarity-based binning, (4) Comparative metagenomics, (5) Functional annotation, (6) Mapping to reference genome

MOCAT usage in the taxonomical estimation of the abundance of reads

As described in Li, Junhua, et al. "An integrated catalog of reference genes in the human gut microbiome." *Nature biotechnology* 32.8 (2014): 834-841, Illumina sequencing reads for fecal samples from European, Chinese and American adults were independently processed (quality control, removal of human sequences, assembling, assembly revision and gene prediction) using MOCAT, which could process metagenomes in a standardized and automated way while improving the quality of assembly and gene prediction compared to using default parameters for the supported programs based on parameter exploration and data-driven parameter optimization at run time.

In addition, MOCAT has been used for estimating taxonomic abundance profiles as described in Wesolowska-Andersen, Agata, et al. "Choice of bacterial DNA extraction method from fecal material influences community structure as evaluated by metagenomic analysis." *Microbiome* 2.1 (2014): 1.

Reference database

The reference database used in the experiment was partially retrieved from the Turnbaugh experiment and completed using the strategy below. It was not possible to establish contact with the original authors. See the Appendix 1 for the full list of genomes.

1. All genomes that appeared in the original paper were considered and selected.
2. A verification step was carried out performing a MEGABLAST search against the GenBank database to make sure the most representative genomes had been selected.

The MEGABLAST execution was performed using 10,000 random reads from each condition (namely Lean and Obese) against the GenBank database. The executions were ran using a word size of 28 and 32 threads. Table 3 shows time details.

Random samples (10,000 reads each)	Average length of reads (base pairs)	Real time
Lean condition	218	71m 12.455s
Obese condition	180	59m 10.567s

Table 3: Runtime executions of the MEGABLAST for the lean and obese 10,000 random samples, using a word size of 28 and 32 threads.

Contrasting signals between a highly abundant taxon and a closely related one

The provided tool *Quality Mapper* in its two types (All vs. all, one vs. all) enables the user to find how reads are being distributed as second best option from a first best candidate, i.e. if genome *g* has 100 mapped reads as first best option, where did the reads match as second best option for those 100 mapped reads? Following the example, up to 100 second best options might be available (or even zero). The lesser the second to third options the better, for it means that the read was matched with more independence and therefore certainty.

For example, calling the script *qmapper1vsAll* with a target genome (say the genome that is a highly abundant taxon) will produce a tabular matrix containing the following:

1. The total mapped reads to the target genome, along with their average identity, length and coverage.
2. From the total of reads in (1), the number of which have been mapped as second best option and to which candidate, showing therefore how these mapped reads “would go to” if there were no first option (as if we removed the highly abundant taxon).

Diseased Metagenome	Hyopneumoniae as 1st best mapping option	Floccurare as 2nd best mapping option
Average Identity	98	85
Average Coverage	99	84
Average Length	479	389
Total reads in the metagenome		677,873
Total reads mapped to Hyopneumoniae as first option		643,243
Total reads where Hyopneumoniae is first and Floccurare is second		323,489

Table 4: A *GMAP* execution between a highly abundant taxon (in the table, Hyopneumoniae) and the closely related one (in the table, Floccurare) shows that out of the 648,988 reads in the metagenome, 643,243 were mapped to Hyopneumoniae, and from those reads, 323,489 would have been mapped to Floccurare if there was no Hyopnemoniae in the sample.

Table 4 shows an interesting result. Although Hyopneumoniae takes up to the 94% of the reads in the sample, more than 50% of their reads are closely related to Floccurare, in the sense that if there was no Hyopnuemoniae in the sample, these would have been mapped to Flocculare in average with 85% identity, 84% coverage and 389 bp length, which are considerable good values for a mapping. On the other side, the averaged values to the ones of the reads mapping to Hyopneumoniae (namely 98% identity, 99% coverage and 479 bp length) are generally better, and can therefore be used to affiance the certainty in the quality of the map.

Handling of blocking organisms

How are reads handled in the scenario where a sample contains only one genome from a species, and the reference database contains two different strain genomes, and the true genome is similar to both in different places (sharing some genes with the one, some with the other)?

The read will be assigned to the best matching genome (let’s say, genome A, g_a) as first matching option, and store all information regarding the second match; let’s say, to genome B, (g_b), as second option.

To all effects, only the match with g_a is accounted as abundance. However, match with g_a (which is NOT included in abundance calculation) is used to estimate how “clear” is the match (distance between matches).

Statistical significance

[Quoted from paper] The presented software can provide statistical data on a number of aspects or characteristics, such as the Z-score test to detect significant variations in the abundance of species in different experimental conditions; or to contrast the significance of the variation at a species level between samples calculating the p-values. An interesting example is case-control studies in which differences in reads abundance along genomes can be identified. Z-scores provide accurate information on the significance of such differences (see “Statistical Significance” in the Supplementary Material for more information). [Quoted from paper]

The Z-score test enables to observe significant variation between samples. In the case of Figure 1, the Z-score test was computed over the reads abundance, showing which genomes are under-expressed (close to zero standard deviations) and those that are over-expressed (above 2~3 standard deviations).

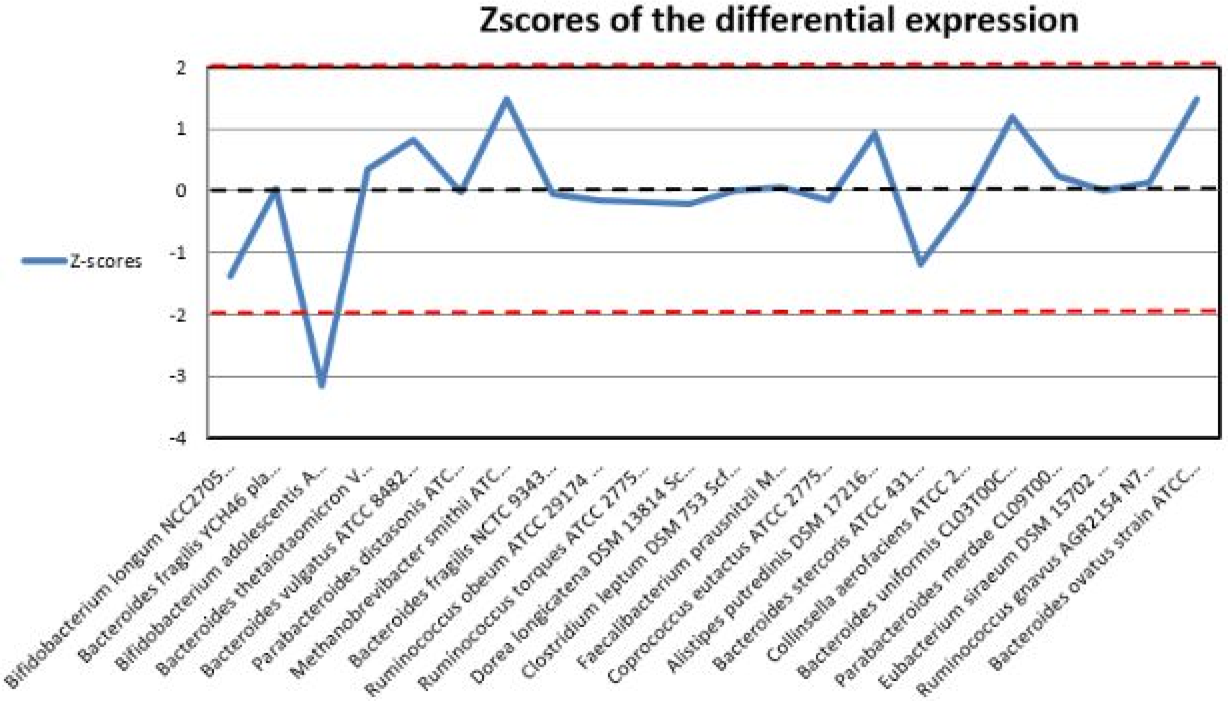

Figure 1: Plot showing a traditional Z-score test . Red dashed lines represent a statistical threshold of significance (above or below 2 standard deviations). The black dashed line shows no deviation at all. In this example, only one species, namely Bacteroides adolescentis shows a significant abundance variation.

Reads mapping to specific regions of genomes

[Quoted from paper] Besides the proximity measures provided by three-option mapping, there is another important aspect concerning the provision of evidence about the presence of species with low-abundance of reads in the metagenome. The main idea is to find regions in a particular organism that do not exist or do not share similarity at all with other organisms present in the collection of genomes. To accomplish this, N-1 comparisons between the reference genome and the N genomes contained in the collection are performed using GECKO. This process yields the detected regions and the assigned reads that have been mapped to these regions.

The extracted reads mapped to these regions provide strong evidence on the presence of low-abundance species in the metagenomic sample, since the mapped read does not fit over other genomes (see “Reads mapping to specific regions of genomes” Supplementary Material for more information). [Quoted from paper]

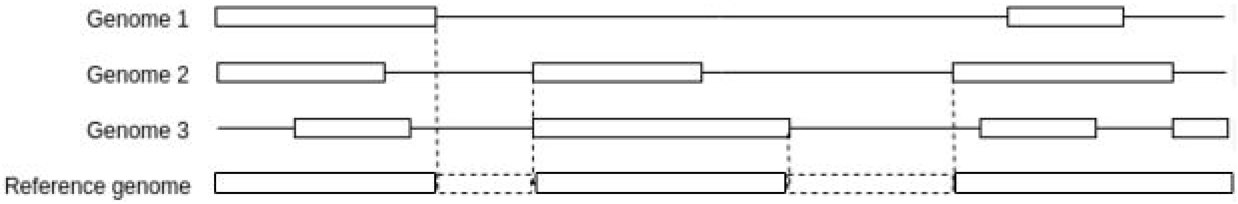

Figure 2: Example of specific regions detected in a database with $N = 4$. Each horizontal line represents a genome. They do not need to be necessarily of the same length, they are showed in the picture in order to facilitate its understanding. Each rectangle (but the dotted ones) represents a fragment shared between the genome of the line where it is present and the reference genome. The dotted rectangles represent the specific regions of the reference genome, where clearly there are no fragments in any of the genomes.

Visual exploration of metagenome distributions

The distribution of matching reads used in the previous section is displayed now in 3D. The main reason is to show a particular behaviour we found in all the experiments we have carried out until now. Sander and Rost curves were obtained for genes and proteins. Currently we are working at genome level in which different types of matches can be observed. Clearly, we identify reads matches at high level of similarity, most of them corresponding to annotated regions of genomes, and a second group of matches with lower length and lower similarity. We have verified most of these second type of matches belongs to non-annotated regions. Therefore using a single threshold as the Sander and Rost curves can derivate in noise or a high number of unassigned reads.

We offer the possibility to custom exploration of distributions, plotted in two or three dimensions using any parameters, such as length, identity, coverage, etc. These distributions can be, for example (1) Metagenome or raw reads by length. (2) The fragments reported by BLAST or GECKO, by length, identity and accumulation. (3) Single distribution for a particular bacteria in the database, which allows to compare a bacteria's distribution with the whole distribution of the metagenome or another bacteria. (4) The distribution for the distinct types of options for a particular bacteria, such as only show those that were second candidates for matches against another subject bacteria.

The stored information in datafiles allows for many combinations of results, which can be later plotted to help visualisation. The two suggested distributions could be later on used in the mapping process assign different levels of value to reads depending on the distribution to which they belong.

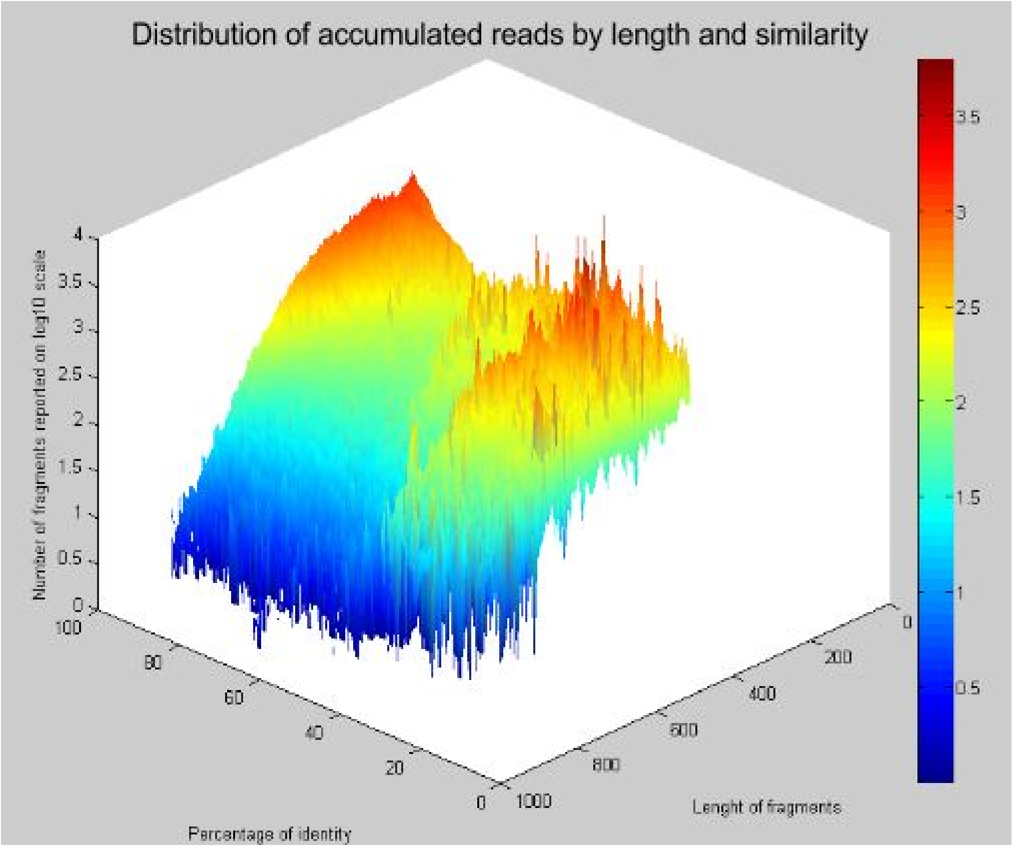

Figure 3: Example of the exploration of the joined distribution of two metagenomes, one healthy and another diseased, by length of reported fragments and percentage of identity, and the altitude showing the accumulation. Please note that for the sake of simplicity, the length axis was inverted to be able to show both sides of the distribution. This plot suggests two natural accumulations which resemble normal distributions.

Coverage curves

Another result of interest is the possibility to extract and plot curves of chosen aspects for all the distributions mentioned before (exploration of distributions). Examples of such results would include (1) Showing the averaged percentage of identity along a custom interval of length, coverage, etc. for a single bacteria, some bacteria or the whole reference database. (2) Plotting a coverage curve to show how coverage varies between certain lengths for particular bacterias. (3) Show differences between strains belonging to the same bacteria in terms of percentage of identity or coverage. As before, the storing system allows for many combinations of not only assigned reads, but also candidate reads, close candidates (using custom parameters) and comparing them against single, some or all organisms.

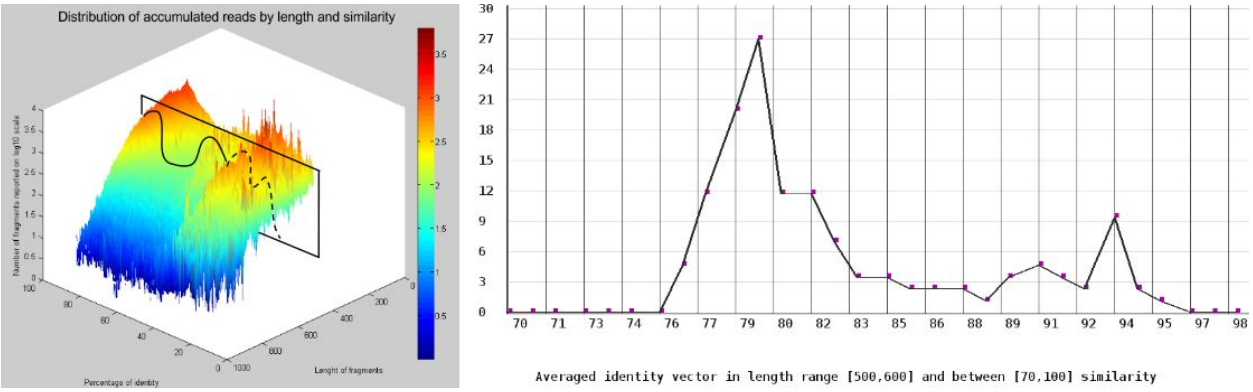

Figure 4: Illustration that shows the accumulation of assigned reads between custom length (in this case, 500 bp and 600 bp) and similarity (percentage of identity from 70% to 100%) for a single organism considering only the best candidates for the map, in particular, Weeksella Virosa. The plot shows that most of the reads tend to accumulate between 76% and 80% of identity. These reads could be extracted and taken into account for analysis since evidence of presence becomes more strong as the number of reads increase for a particular region.

As we mention in the previous sub-section, the distribution of fragments in the case of short sequence versus full-genome depicts a binomial distribution (see Figure 3 and 4). This is a strong recommendation for taking different thresholds during the mapping process.

Taxonomy files

A taxonomical description file allows for custom boundaries and relationships between organisms in the reference database, such as imposing some species to be strain related, or to separate strains that belong to a common ancestor. Such file can be generated automatically using an included module in the workflow and/or can be manually built to insert custom relations between species. The used format consists of a text file including a 5-tuple per line (length not included in table 5), each tuple being a new genome, which can be a full species or a subspecies. The key point is to represent scaffolds and contigs as substrains of the first one.

Species Level 1	Species Level 2	Access code	Species Name
1	0	ref|NC_021831.1|	Mycoplasma hyopneumoniae J
1	1	ref|NC_017509.1|	Mycoplasma hyopneumoniae 168
2	0	ref|NC_015144.1|	Weeksella virosa DSM 16922
3	0	ref|NZ_DS264342.1|	Ruminococcus obeum ATCC 29174 Scfld0253
3	1	ref|NZ_DS264341.1|	Ruminococcus obeum ATCC 29174 Scfld0254
3	2	ref|NZ_DS264340.1|	Ruminococcus obeum ATCC 29174 Scfld0255

Table 5: Example of taxonomical description file where two species are bounded together as substrains using the same number for Species Level 1, a second species has no substrain attached and a third species composed of three contigs. Such added information will allow in later steps more refined mapping results.

About the parsing format in the workflow

In order to use any comparison software such as BLAST, GECKO, LASTz or others, a common format was introduced to which any of these software comparison results should be parsed to. The format consists in a tab based text file composed of a header with one or more 12-tuples.

A header, one for each unique combination of *read* and *genome,* along with *length*:

>Read ID	>Genome access number and name	Genome length

Table 6: Header description of the parsing format

Multiple fragments, as many as reported for the tuple, keeping the following fields:

$$t^{12}_{n,k} = (k, score, expected, identities, length, similarity, igaps, egaps, strand, xStart, xEnd, yStart, yEnd)$$

Where each field corresponds to:

Field	Description
Fragment number	For every read, the position in the list of reported fragments
Score	Reported score for the fragment
Identities	Number of identities
Matching length	Length of the match
% Similarity	The number of identities divided by the matching length
iGaps	Number of opening gaps
eGaps	Number of extension gaps
Strand	Strand of the fragment represented with ++ or +-
Start in read	Starting position of the match in the read coordinates
End in read	Ending position of the match in the read coordinates
Start in genome	Starting position of the match in the genome coordinates
End in genome	Ending position of the match in the genome coordinates

Table 7: Detailed description of a tuple representing a match

```
>READ ID >GENOME CODE >GENOME LENGTH
(FRAG ID) (SCORE) (IDENTITIES) (LENGTH) (SIMILARITY) (IGAPS) (EGAPS) (STRAND)
(READ START) (READ END) (GENOME START) (GENOME END)
```

```
>H8T5UUH01DRDOX >gi|71893359|ref|NC_007295.1| 897405
1    44.1     31    33    94      2     0    ++    35   65    871680   871712
2    40.1     20    20    100     0     0    ++    1    20    871637   871656
```

Table 8: (UP) A header with a tuple displaying the contents of each field. (DOWN) A read is shown with two reported fragments.

Details of the extension of ungapped HSPs

In most cases metagenomes will be composed of millions of reads, which consequently will have hundreds of millions of reported aligned fragments. In order to process this large amount of data in reasonable time, sorted index are created to allow fast load and search.
The database is fully loaded into memory, as genomes are large and require more disk drive access time. Loaded genomes are accessed via an accession number index using binary search. Reads are kept in disk to be loaded using a sorted index again with binary search.

1. Load genomes into memory
2. Quicksort genomes by accession number
3. Load reads index
4. For every read in the comparison file
    a. Sort all fragments belonging to the read by read coordinates
    b. For all tuples of consecutive fragments $(f_n f_{n+1})$
        i. If $(f_n f_{n+1})$ distance in both genome and read coordinates satisfy the threshold
            1. Load the complete read using binary search
            2. Perform Needleman-Wunsh scoring matrix between $(f_n.r_1 f_{n+1}.r_2)$ and $(f_n.g_1 f_{n+1}.g_2)$
            3. If the obtained alignment satisfies minimum coverage and similarity
                a. Join fragments
    c. Store in output file

Notice that for a *glocal* alignment the cost of extending a gap should be zero or close, to allow a "floating" fragment.

Working example

Say we have the following three reported fragments for the tuple (`R`, `gi|71893359|`):

```
>R >gi|71893359|ref|NC_007295.1| 897405
1    44.1    31    33    94     2    0    ++    35    65    871680    871712
2    40.1    20    20    100    0    0    ++    1     20    871637    871656
3    30.2    15    15    100    0    0    +-    17    31    68482     68468
```

Table 9: Example of candidate reported fragments

As we can see, the two fragments marked in green represent a chance to join them, since:

1. Both share the same strand.
2. The separation between fragments in read (15 bp) and genome (24 bp) coordinates is less than the given maximum (say we chose a maximum separation of 50 bp).

Then we proceed to extract the read and the region within the genome:

Read region	TGCTGTTAGATGTCTATATTATCAAGTTAGATTCACTGCTCTTATAATCTTCTTCTGAGAAGATTGATCT
Genome region	TGCTGTTAGATGTCTATATTTATCAAAAAGTTTAAGATTTTCAACTGCTCTTATAAAATCTTCTTCTGAGAAGATT

Table 10: Reported fragments within the read and genome sequences

Reported matches for the fragment are colored in light blue and green.

Since we do not need to store the alignments, and in the sake of speed, only the resulting matching length, number of identities and gaps is considered. Thus, the in-depth alignment between the two sequences produces the following joined fragment:

```
>R >gi|71893359|ref|NC_007295.1| 897405
1     NULL     57     65     87      5     6     ++     1     65     871637     871712
1     44.1     31     33     94      2     0     ++     35    65     871680     871712
2     40.1     20     20     100     0     0     ++     1     20     871637     871656
3     30.2     15     15     100     0     0     +-     17    31     68482      68468
```

Table 11: In red, removed fragments that have been joined to conform the green fragment.

The joined fragments are inserted as a new one, while their remains are removed.

The new fragment has 57 identities out of a 65 bp length match, meaning an 87% similarity. Small variations in the Needleman-Wunsch parameters won't modify the aligned outcome excessively. Please notice that the scores are set to NULL since a new score will be calculated prior to the mapping.

As a punctual note, if we blast the two sequences using Blast's Online Nucleotide Alignment Tool BlastN, we obtain quite similar results, from which variations are due to different parameters:

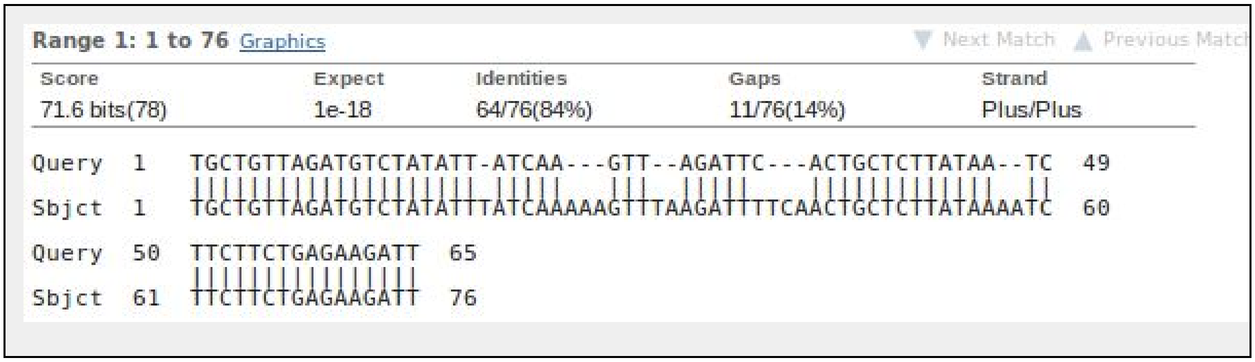

Figure 5: Results of blasting the two sequences above using Blastn and default parameters.

Calculating the score and expected values after the extension

The procedure to calculate new scores and expected values follows a similar procedure to the one in BLAST, using Karlin and Lambda parameters. The whole method consists of the following steps:

1. Calculate the raw score for every fragment. To compute the raw score of the extended fragment produced by our custom glocal alignment we apply a traditional affine scoring model (with open and extension gap penalties) the following formula:

$$RS = I * Mr + (L - (Gi + Ge) - I) * Mp + Gi * Pi + Ge * Pe$$

   Where RS stands for “Raw Score”, I for the total number of identities in the fragment, Mr for the match score, L for the total length of the fragment in base pairs, Gi for the total number of open gaps in the fragment, Ge for the total number of extension gaps in the fragment, Mp for the mismatch penalty, Pi for the penalty of an open gap and Pe for the penalty of an extension gap.

2. A bitscore is calculated from the rawscore using Karlin and Lambda parameters, which is the normalized score:

$$S' = \frac{\lambda S - \ln K}{\ln 2}$$

Where S' is the bitscore, S the rawscore, K is the Karlin parameter and λ is the Lambda parameter.

3. For the last step, the expected value is calculated as:

$$E = Kmn\, e^{-\lambda S}$$

Where m is the length of the reported fragment and n is the length of the target genome divided by the total number of residues in the database.

Mapping decision and fragments

This section will illustrate an example of mapping for a list of fragments belonging to different reads. Consider Table 12:

>Read 1 >Genome X										
1	0	134	135	99	0 0	+-	1	134	3833768	3833901
2	0	30	30	100	0 0	+-	136	164	3833902	3833930
3	0	48	48	100	0 0	+-	167	213	3833932	3833978
>Read 1 >Genome Y										
1	0	82	135	60	0 0	++	1	134	4422641	4422774
2	0	193	202	95	0 0	+-	1	201	1094845	1095045
>Read 2 >Genome X										
1	0	20	21	95	0 0	++	1	20	453108	453127

Table 12: Example of parsed fragments that are going to be mapped.

Table 12 shows two reads (*Read 1* and *Read 2*), which are matched to genomes *X,Y* and *X*, respectively. The first step is extracting only those fragments belonging to *Read 1*. The list of reported fragments for *Read 1* are:

>Read 1										
>Genome X										
1	0	134	135	99	0 0	+-	1	134	3833768	3833901
2	0	30	30	100	0 0	+-	136	164	3833902	3833930
3	0	48	48	100	0 0	+-	167	213	3833932	3833978
>Genome Y										

1	0	82	135	60	0 0	++	1	134	4422641	4422774
2	0	193	202	95	0 0	+-	1	201	1094845	1095045

Table 13: List of reported fragments for *Read 1*.

The first phase is the coverage filtering. Using an index for the reads, the mapping module retrieves the length of *Read 1,* which is 200 nucleotides long. Thus the coverage filtering will discard those fragments that are below the coverage thresholds (say, 50% for this example).

>Read 1										
>Genome X										
1	0	134	135	99	0 0	+-	1	134	3833768	3833901
~~2~~	~~0~~	~~30~~	~~30~~	~~100~~	~~0 0~~	~~+-~~	~~136~~	~~164~~	~~3833902~~	~~3833930~~
~~3~~	~~0~~	~~48~~	~~48~~	~~100~~	~~0 0~~	~~+-~~	~~167~~	~~213~~	~~3833932~~	~~3833978~~
>Genome Y										
1	0	82	135	60	0 0	++	1	134	4422641	4422774
2	0	193	202	95	0 0	+-	1	201	1094845	1095045

Table 14: List of reported fragments for *Read 1* after the coverage filtering phase. In green, the length of the match, used to calculate the coverage of the fragment. In red, the inactivated fragments.

Now the identity filtering phase inactivates all fragments whose percentage of identity is below the threshold (say, 80% for this example).

>Read 1										
>Genome X										
1	0	134	135	99	0 0	+-	1	134	3833768	3833901
>Genome Y										
~~1~~	~~0~~	~~82~~	~~135~~	~~60~~	~~0 0~~	~~++~~	~~1~~	~~134~~	~~4422641~~	~~4422774~~
2	0	193	202	95	0 0	+-	1	201	1094845	1095045

Table 15: List of reported fragments for *Read 1* after the coverage and identity filtering phase. In green, the percentage of identity. In red, inactivated fragments.

Now the 3-option mapping is performed. The fragment with the smallest expected value is selected (calculated as explained in the section before "*Calculating the score and expected value after the extension"*). Say the fragment belonging to *Read 1* aligned to *Genome Y* has an expected value smaller than that of *Read 1* aligned to *Genome X*. Then that fragment, *Read 1* aligned to *Genome Y* is set as the first option for *Read 1*, and inactivated for the next iteration. In the next iteration, only one fragment, *Read 1* aligned to *Genome X* is left, and will therefore be selected as the second best option. No third option will be available, and thus *Read 1* will only have two possible options.

Comparison with MEGAN 5

[Quoted from paper] In order to prove that the results of the proposed workflow are consistent with those of other metagenomic analysis software suites (in terms of abundance in the taxonomic classification), the following test was performed using results from BLASTn based on metagenomic samples from faecal microbial communities. Both, MG workflow and MEGAN were executed using the same input from BLASTn and ran with default parameters (available in the Supplementary Material under "*Comparison with MEGAN"*).

On comparison of the lean metagenome based on MEGAN, the abundance plot (See Figure 4) shows similar results to ours. Standard deviation from ratios (using abundance data provided by MEGAN and by our workflow) was 0.25, which is not significant enough to identify relevant variations. However, whereas the analysis of a metagenome using MEGAN can last nearly an hour, our MG workflow took about six minutes to analyze the obese metagenome and five minutes for the lean one when BLAST the comparison had been done with BLAST. With GECKO, the duration of the process was further reduced, taking about only one minute for the lean sample and three minutes and a half for the obese metagenome. Runtime executions were measured using a standard Intel i5 machine with 4GB of RAM.[Quoted from paper]

Both mapping modules were executed using BLASTn's output and therefore the only differences in the reads abundance is due to mapping particularities, thus we will only mention the parameters of the mapping module.

For the execution and fair comparison, default parameters both MEGAN and MG workflow were used. Since filtering modes are different (i.e. minimum score versus coverage and similarity thresholds) and the way expected values are calculated are not exactly the same, it becomes difficult to find a complete and absolute equivalence between parameters. The table below shows the relative parameter equivalence and the reasons behind the equivalence:

MEGAN 5 parameters	MG Workflow parameters	Comments
Minimum Score 30	Minimum coverage threshold 40%<br>Minimum identity threshold 40%	(*)
E-value 0.01	E-Value 0.01	Same parameter. However, the calculation of the e-value is not absolutely equivalent as BLASTn's.

Table 16: Comparison of the default parameters of MEGAN and MG workflow.

(*) The score depends on the length of the alignment, the length of the database, the number of

identities and the number of gaps. A minimum identity threshold of 40% is default and actually almost non-restrictive, since the reported fragments of any sequence comparison software filters up to 70% of identity. However, we allow these fragments (in case they exist) to have a second chance on the extension step which might increases their identity value. On the other side, a minimum threshold of 40% coverage implies that small fragments according to the length of the read will be filtered, as these will be considered spurious matches. The combination of the two filters (identity and coverage) result in a similar filtering way as a score threshold, but allows for more customization.

Core-datafile definitions

Mapping structure

The core storing datafile holds the information regarding a read and its mapping properties. Each mapped read will have at least one *mapRG* struct, and as many as three for a three-options mapping.

```
// Read-Genome mapping structure
struct mapRG {
    int    rNumber;      // Read number in the original metagenome file
    int    gNumber1;     // Genome number level 1 - Species level
    int    gNumber2;     // Genome number level 2 - Subspecies level
    int    gPosList;     // Position in the taxonomy file list
    double score;        // Obtained score (if any)
    int    igaps;        // Number of opening gaps
    int    egaps;        // Number of extension gaps
    int    lengthGapped; // Length in nucleotides if considering different alignments
    float  expected;     // Expected value (if any)
    int    nIdent;       // Number of identities
    int    matchLen;     // Matching length of the alignment
    int    pIdent;       // Percentage of identity
    char   strand[MAXLID];   // Strand [++]||[+-]
    int    rStart, rEnd; // Read matching coordinates
    int    gStart, gEnd; // Genome matching coordinates
    int    option;       // Mapping options
    int    coverage;     // Coverage of the match for the read
};
```

The structure can be easily extended with new fields to allow new processing steps.

Gene structure

Another structure is needed to represent annotated regions, which will be used in some of the post-processing programs.

```
struct annotation{
    int start;      // Starting position in the genome coordinates
    int end;        // Ending position
    char strand1;
    char strand2;
    char tag[MAXLID];   // Tag of the annotated region
    char annotation[MAXLID];    // Text field to hold any property such as 'product'
};
```

All structs are meant to be expanded to fulfil new on-demand experiments.

Genome-representation structure

This structure is used to relate length and strains during mapping, and to allow using the genome's names in output files.

```
struct GenomeList {
    int species;
    int subSpecies;
    char id[MAXLID];
    char name[MAXLID];
    int len;
};
```

Post-processed-datafile definitions

All processed results are generated from the mapping results, some in addition with annotation files. These are all aimed to be easy to use text-based files that can be edited, reviewed or exploited using external software such as spreadsheets or script-like programming frameworks (such as Matlab or R).

Mapping results and the abundance file

The mapping process provides with not only the binary files which hold the structures with the mapping results for every read, but also provides two texts files with the results about abundance in the different options, total mapped reads, shared reads, etc. These files should be

opened as if they were spreadsheets, since they use tab-based format. One text file displays the results per every genome in the database (this is, per species and subspecies) and the other displays aggregated information of only the species.

	A	B	C	D	E	F	G	H	I	J
1	Matched:	1608491								
2	Unmatched:	1116376								
3	Total Reads:	2724866								
4	G.ID	G.ID2	1st	2nd	3d	Shared	diffIdent	diffCov	diffLength	Genome Code
5	1	0	9936	10961	6282	502	2.36E+00	1.21E+00	1.04E+00	NC_004943.1
6	2	0	9728	30265	78783	3541	8.91E-01	1.73E-01	1.86E-01	NC_006297.1
7	3	0	11062	9782	6011	570	1.99E+00	1.53E+00	1.15E+00	NC_008618.1
8	4	0	47155	128685	73696	6816	1.02E+00	2.90E-01	3.58E-01	NC_004703.1
9	5	0	288028	33085	37347	5074	2.04E+00	6.15E-01	6.35E-01	NC_009614.1
10	6	0	38788	34803	21352	1246	1.97E+00	1.07E+00	9.66E-01	NC_009615.1
11	7	0	9352	5504	4511	741	1.86E+00	3.59E+00	2.41E+00	NC_009515.1
12	8	0	15383	52040	83065	11327	1.11E-01	3.53E-02	3.80E-02	NC_006873.1
13	9	0	144692	60459	35000	14356	1.25E-01	1.10E-01	1.02E-01	NZ_DS264289.1
14	10	0	54584	49736	37954	6402	1.55E-01	1.92E-01	1.72E-01	NZ_DS264343.1
15	11	0	95867	65930	39453	11961	1.93E-01	1.59E-01	1.52E-01	NZ_DS264384.1
16	12	0	34450	35366	20571	2608	3.12E-01	5.25E-01	3.43E-01	NZ_DS480331.1
17	13	0	173175	26139	15218	5522	2.29E-01	2.16E-01	1.67E-01	NZ_DS483479.1
18	14	0	49717	38646	28537	4682	2.87E-01	3.16E-01	2.54E-01	NZ_DS483520.1
19	15	0	70654	11456	8870	2289	2.60E-01	2.85E-01	2.13E-01	NZ_DS499570.1
20	16	0	64294	137985	58277	22431	2.10E-01	1.78E-01	1.90E-01	NZ_DS499661.1
21	17	0	22058	15715	9579	1650	3.72E-01	1.06E+00	6.15E-01	NZ_AAVN02000023.1
22	18	0	179530	64597	53253	5869	2.50E-01	7.43E-02	7.52E-02	NZ_JH724267.1
23	19	0	54131	58253	29084	6948	2.94E-01	2.64E-01	2.36E-01	NZ_JH976534.1
24	20	0	37461	20674	15389	2927	1.72E-01	2.53E-01	1.80E-01	NZ_KB907545.1
25	21	0	59003	60681	47084	2601	1.08E+00	1.32E+00	8.67E-01	NZ_JAGQ01000005.1
26	22	0	139443	73911	62688	6545	2.58E+00	6.70E-01	7.74E-01	NZ_CP012938.1

Figure 6: Generated abundance file per species only opened using any spreadsheet software.

Mainly two things are shown:

1. A header with the total number of matched (mapped) reads, the unmatched (no candidate or no candidate good enough given the mapping parameters) and the total reads present in the metagenome.
2. The information regarding the mapping results for all species (also the file with subspecies level is generated).

The following table explains the meaning and value of each column in the abundance file:

**Column**	**Meaning**
A	Genome species ID as indicated in the taxonomy file
B	Genome subspecies ID as indicated in the taxonomy file
C	Number of mapped reads in a specific genome (namely First Option)
D	2nd match option (the second match is computed only over the species that are not substrains of the 1st mapped option)

E	3^d match option (the third match is computed only over the species that are not substrains of the 1st and 2nd mapped option)
F	The number of reads (of the first option, this is, total maps for a genome) that have been labeled as “close” to the second option (i.e. the second best option for a genome was between 10 bp shorter in the alignment and had 5 identities less)
G, H, I	For those reads that are shared, the average difference in identities (number), coverage (percentage) and matching length (number) between the first option and the close option.
J	Species reference codes. If using the per species abundance file only the first reference code is shown.
K	(Not shown in the image) Name of the species

Table 17: Details of the meaning of every column of the mapping results produced by GMAP.

Genbank parsed datafiles

The parsing system for GenBank files extracts only the needed fields for the current post-processing modules. Again, the parsing system is capable of being expanded to include new fields and parameters.

Gene start	Gene end	Strand	(reserved)	Locus tag	Product
687	3158	f	-	MHO_RS00005	membrane protein insertase YidC

Table 18: Extracted fields and parsed format for an annotation file.

Above, an example of the Genbank parsing system showing which fields are being stored.

Genome accumulated profiles

The accumulated profiles for particular genomes consist of a two-column text file containing the genome consecutive positions and the number of accumulated nucleotides per each of these positions.

Position	Accumulated nucleotides
1	0
2	3
3	12

4	11
5	14
...	...

Table 19: Representation of a genome profile

Matrix files

MAT files are used to represent distributions of any type (such as length by identity, or coverage) of whole distributions or particular genomes for which the experiment was carried out. Many modules in the workflow use this particular file format, for example, the *quality mapper*, which is the program used to extract averaged differences between first and other options.

Genome Id	First option total maps	First option identity average	First option average coverage	First option average length	Second option total maps	...
0	0	0	0	0	0	...
1	0	0	0	0	620	...
2	0	0	0	0	1895	...
3	0	0	0	0	741	...
4	0	0	0	0	1388	...
5	0	0	0	0	831	...
6	0	0	0	0	609	...
7	0	0	0	0	1539	...
8	0	0	0	0	1583	...
9	385195	87	38	79	0	...
...	...	...	...	...	...	...

Table 20: Example of MAT file containing a one-versus-all comparison showing that genome 9 was compared against the whole database.

Workflow extension and re-implementation example

In order to illustrate how to use the binary mapping files for further and custom extension of the workflow, and also to provide insight on how binary structures are used, a simple exercise is proposed. We will use the C programming language to load data from the binary mapping file, to process it and to finally produce our own data.

It will be assumed that the user has already performed an experiment and therefore owns binary mapping files. If not, we absolutely encourage the user to try our *Guided Exercises*, which show how to easily execute the whole workflow via the Galaxy web interface. Please find it at http://chirimoyo.ac.uma.es/gecko.

Download and gather the code

At the same location as the *Guided Exercise*, you will find the binaries for the workflow. Another tutorial is available to get you through the installation at http://chirimoyo.ac.uma.es/gecko. We will assume installation was already done.

Open a terminal and navigate to your METAGECKO workflow folder, and then enter the *bin* directory.

Creating a new module

In this example we will code a program that will load data from disk (from the binary mapping files), extract some information (length of the mapped reads, for example) and finally print it on screen. We will code a simple and basic program that calculates the average length of mapped reads.

You can create a new text document using any software you wish: Gedit, vim, Sublime… In this case, we will use Gedit, as it is installed in almost every known unix machine. Type in the following:

```
gedit mapped_reads_len.c &
```

The command will open Gedit and run it in background. Now we will start coding.

Simple template

We suggest you to use the following template which will help you start coding:

```
/*

@Filename
@Author
@Description

@Input parameters

```

```
@Output files


*/


#include <stdlib.h>
#include <stdio.h>
#include "funcMG.h"

int main(int argc, char **argv){
    return 0;
}
```

Adding some basic code

The full code, commented line by line is available at the end of this document. We will review only the code sentences of interest.

The following code opens a binary mapping file stored on disk:

```
    //Create file to load data
    FILE * binary_map = NULL;
    //Read as binary
    binary_map = fopen64(av[1], "rb");
```

Now we need the particular data structure to read from the mapping file:

```
    //Create a struct to read the binary mapping file
    struct mapRG mRG;
```

Adding some variables to save read length accumulation:

```
    //Variable to store the average length, read counter and total sum
    long int sum = 0;
    long int num_reads = 0;
    double average = 0.0;
```

Then, code a main loop to sweep the whole mapping file, from the first to last match:

```
    while(!feof(binary_map)){ //Until we have read the complete file
       //Read one map assignment
       fread(&mRG, sizeof(struct mapRG),1,binary_map);
       //ADD CODE HERE
    }
```

In the *ADD CODE HERE* section, you can add any processing you may want. For this example we will be checking if the matched read is a first option (best candidate), and if so, account for its length.

```
   //..
       //Consider only those reads that are the best candidate
       if(mRG.option == 1){
             //Increase num_reads and add length
             num_reads++;
             sum += mRG.matchLen;
       }
   //..
```

Now print the data in a regular way, or maybe store it in a file:

```
    //Print gathered data
    fprintf(stdout, "Total number of reads matched as first option: %Ld\n",
num_reads);

    //Compute the average length and print it on screen
    if(num_reads > 0){
       average = (double)sum/num_reads;
       fprintf(stdout, "The average length of the matched reads is %e. (%d
nucleotides, rounded).\n", average, (int) average);
    }else{
       fprintf(stdout, "Read number is zero.\n");
    }
```

Finally close the binary map file:

```
    fclose(binary_map);
```

And that’s about it. Writing a simple program that interacts with the results of the mapping process is simple and can be extended to fulfill further experiments with custom processing.

Compiling and executing

Now that we are done with the code, we are ready to compile (and hope for the best!) and run. To compile the source files, use:

```
gcc mapped_reads_len.c funcMG.c -D_FILE_OFFSET_BITS=64
-D_LARGEFILE_SOURCE -D_LARGEFILE64_SOURCE -o mapped_reads_len -w
```

This will produce the executable “mapped_reads_len”. Now we can run it by typing:

```
./mapped_reads_len <binary_file>
```

Running the new module on a small dataset, we get:

```
>./mapped_reads_len my_binary_file.mapbin
Total number of reads matched as first option: 110304
The average length of the matched reads is 1.063660e+02. (106
nucleotides, rounded).
>
```

Full code available:

```
/*

@Filename
@Author
@Description

@Input parameters

@Output files


*/


#include <stdlib.h>
#include <stdio.h>
#include "funcMG.h" //Provides all needed functions

int main(int argc, char **av){

    if(argc != 2){
       //Exit the program if not enough or too many parameters
       printf("ERROR: ./mapped_reads_len <input_mapping_file>\n");
       exit(-1);
    }
```

```
    //Create file to load data
    FILE * binary_map = NULL;
    //Read as binary
    binary_map = fopen64(av[1], "rb");

    //Check it was correctly opened, exit if wrong
    if(binary_map == NULL){
       printf("Could not open binary input file\n");
       exit(-1);
    }

    //Create a struct to read the binary mapping file
    struct mapRG mRG;

    //Variable to store the average length, read counter and total sum
    long int sum = 0;
    long int num_reads = 0;
    double average = 0.0;

    while(!feof(binary_map)){ //Until we have read the complete file
       //Read one map assignment
       fread(&mRG, sizeof(struct mapRG),1,binary_map);

       //Consider only those reads that are the best candidate
       if(mRG.option == 1){
             //Increase num_reads and add length
             num_reads++;
             sum += mRG.matchLen;
       }
    }

    //Print gathered data
    fprintf(stdout, "Total number of reads matched as first option: %Ld\n",
num_reads);

    //Compute the average length and print it on screen
    if(num_reads > 0){
       average = (double)sum/num_reads;
       fprintf(stdout, "The average length of the matched reads is %e. (%d
nucleotides, rounded).\n", average, (int) average);
    }else{
       fprintf(stdout, "Read number is zero.\n");
    }

    //Close binary mapping file
    fclose(binary_map);

    //Exit program
    return 0;
}
```

Appendix 1 - Reference database used in the experiment

Table 21 shows all sequences included in the reference database.

Accesion number	Name	Length (bp)
NC_004307.2	Bifidobacterium longum NCC2705 chromosome, complete genome	2256640
NC_004943.1	Bifidobacterium longum NCC2705 plasmid pBLO1, complete sequence	3626
NC_006347.1	Bacteroides fragilis YCH46 DNA, complete genome	5277274
NC_006297.1	Bacteroides fragilis YCH46 plasmid pBFY46, complete sequence	33716
NC_008618.1	Bifidobacterium adolescentis ATCC 15703 DNA, complete genome	2089645
NC_004663.1	Bacteroides thetaiotaomicron VPI-5482 chromosome, complete genome	6260361
NC_004703.1	Bacteroides thetaiotaomicron VPI-5482 plasmid p5482, complete sequence	33038
NC_009614.1	Bacteroides vulgatus ATCC 8482, complete genome	5163189
NC_009615.1	Parabacteroides distasonis ATCC 8503, complete genome	4811379
NC_009515.1	Methanobrevibacter smithii ATCC 35061, complete genome	1853160
NC_003228.3	Bacteroides fragilis NCTC 9343, complete genome	5205140
NC_006873.1	Bacteroides fragilis NCTC 9343 pBF9343 plasmid complete sequence	36560
NZ_DS264342.1	Ruminococcus obeum ATCC 29174 Scfld0253 genomic scaffold, whole genome shotgun sequence	102399

NZ_DS264341.1	Ruminococcus obeum ATCC 29174 Scfld0252 genomic scaffold, whole genome shotgun sequence	1067
NZ_DS264340.1	Ruminococcus obeum ATCC 29174 Scfld0251 genomic scaffold, whole genome shotgun sequence	459251
NZ_DS264339.1	Ruminococcus obeum ATCC 29174 Scfld0250 genomic scaffold, whole genome shotgun sequence	183429
NZ_DS264338.1	Ruminococcus obeum ATCC 29174 Scfld0249 genomic scaffold, whole genome shotgun sequence	433990
NZ_DS264337.1	Ruminococcus obeum ATCC 29174 Scfld0248 genomic scaffold, whole genome shotgun sequence	33460
NZ_DS264336.1	Ruminococcus obeum ATCC 29174 Scfld0247 genomic scaffold, whole genome shotgun sequence	147259
NZ_DS264335.1	Ruminococcus obeum ATCC 29174 Scfld0246 genomic scaffold, whole genome shotgun sequence	267
NZ_DS264334.1	Ruminococcus obeum ATCC 29174 Scfld0245 genomic scaffold, whole genome shotgun sequence	2176
NZ_DS264333.1	Ruminococcus obeum ATCC 29174 Scfld0244 genomic scaffold, whole genome shotgun sequence	850
NZ_DS264332.1	Ruminococcus obeum ATCC 29174 Scfld0243 genomic scaffold, whole genome shotgun sequence	3612
NZ_DS264331.1	Ruminococcus obeum ATCC 29174 Scfld0242 genomic scaffold, whole genome shotgun sequence	3398
NZ_DS264330.1	Ruminococcus obeum ATCC 29174 Scfld0241 genomic scaffold, whole genome shotgun sequence	4685
NZ_DS264329.1	Ruminococcus obeum ATCC 29174 Scfld0240 genomic scaffold, whole genome shotgun sequence	6188
NZ_DS264328.1	Ruminococcus obeum ATCC 29174 Scfld0239 genomic scaffold, whole genome shotgun sequence	151737
NZ_DS264327.1	Ruminococcus obeum ATCC 29174 Scfld0238 genomic scaffold, whole genome shotgun sequence	8536
NZ_DS264326.1	Ruminococcus obeum ATCC 29174 Scfld0237 genomic	11349

	scaffold, whole genome shotgun sequence	
NZ_DS264325.1	Ruminococcus obeum ATCC 29174 Scfld0236 genomic scaffold, whole genome shotgun sequence	11444
NZ_DS264324.1	Ruminococcus obeum ATCC 29174 Scfld0235 genomic scaffold, whole genome shotgun sequence	13608
NZ_DS264323.1	Ruminococcus obeum ATCC 29174 Scfld0234 genomic scaffold, whole genome shotgun sequence	17887
NZ_DS264322.1	Ruminococcus obeum ATCC 29174 Scfld0233 genomic scaffold, whole genome shotgun sequence	21219
NZ_DS264321.1	Ruminococcus obeum ATCC 29174 Scfld0232 genomic scaffold, whole genome shotgun sequence	21526
NZ_DS264320.1	Ruminococcus obeum ATCC 29174 Scfld0231 genomic scaffold, whole genome shotgun sequence	16969
NZ_DS264319.1	Ruminococcus obeum ATCC 29174 Scfld0230 genomic scaffold, whole genome shotgun sequence	17999
NZ_DS264318.1	Ruminococcus obeum ATCC 29174 Scfld0229 genomic scaffold, whole genome shotgun sequence	32663
NZ_DS264317.1	Ruminococcus obeum ATCC 29174 Scfld0228 genomic scaffold, whole genome shotgun sequence	33452
NZ_DS264316.1	Ruminococcus obeum ATCC 29174 Scfld0227 genomic scaffold, whole genome shotgun sequence	53002
NZ_DS264315.1	Ruminococcus obeum ATCC 29174 Scfld0226 genomic scaffold, whole genome shotgun sequence	58012
NZ_DS264314.1	Ruminococcus obeum ATCC 29174 Scfld0225 genomic scaffold, whole genome shotgun sequence	63106
NZ_DS264313.1	Ruminococcus obeum ATCC 29174 Scfld0224 genomic scaffold, whole genome shotgun sequence	29161
NZ_DS264312.1	Ruminococcus obeum ATCC 29174 Scfld0223 genomic scaffold, whole genome shotgun sequence	78235
NZ_DS264311.1	Ruminococcus obeum ATCC 29174 Scfld0222 genomic scaffold, whole genome shotgun sequence	217646

NZ_DS264310.1	Ruminococcus obeum ATCC 29174 Scfld0221 genomic scaffold, whole genome shotgun sequence	87451
NZ_DS264309.1	Ruminococcus obeum ATCC 29174 Scfld0220 genomic scaffold, whole genome shotgun sequence	39433
NZ_DS264308.1	Ruminococcus obeum ATCC 29174 Scfld0219 genomic scaffold, whole genome shotgun sequence	55419
NZ_DS264307.1	Ruminococcus obeum ATCC 29174 Scfld0218 genomic scaffold, whole genome shotgun sequence	119753
NZ_DS264306.1	Ruminococcus obeum ATCC 29174 Scfld0217 genomic scaffold, whole genome shotgun sequence	122734
NZ_DS264305.1	Ruminococcus obeum ATCC 29174 Scfld0216 genomic scaffold, whole genome shotgun sequence	69937
NZ_DS264304.1	Ruminococcus obeum ATCC 29174 Scfld0215 genomic scaffold, whole genome shotgun sequence	118432
NZ_DS264303.1	Ruminococcus obeum ATCC 29174 Scfld0214 genomic scaffold, whole genome shotgun sequence	150462
NZ_DS264302.1	Ruminococcus obeum ATCC 29174 Scfld0213 genomic scaffold, whole genome shotgun sequence	55225
NZ_DS264301.1	Ruminococcus obeum ATCC 29174 Scfld0212 genomic scaffold, whole genome shotgun sequence	29419
NZ_DS264300.1	Ruminococcus obeum ATCC 29174 Scfld0211 genomic scaffold, whole genome shotgun sequence	134
NZ_DS264299.1	Ruminococcus obeum ATCC 29174 Scfld0210 genomic scaffold, whole genome shotgun sequence	814
NZ_DS264298.1	Ruminococcus obeum ATCC 29174 Scfld029 genomic scaffold, whole genome shotgun sequence	112072
NZ_DS264297.1	Ruminococcus obeum ATCC 29174 Scfld028 genomic scaffold, whole genome shotgun sequence	112359
NZ_DS264296.1	Ruminococcus obeum ATCC 29174 Scfld027 genomic scaffold, whole genome shotgun sequence	58637
NZ_DS264295.1	Ruminococcus obeum ATCC 29174 Scfld026 genomic	11505

	scaffold, whole genome shotgun sequence	
NZ_DS264294.1	Ruminococcus obeum ATCC 29174 Scfld025 genomic scaffold, whole genome shotgun sequence	52960
NZ_DS264293.1	Ruminococcus obeum ATCC 29174 Scfld024 genomic scaffold, whole genome shotgun sequence	2412
NZ_DS264292.1	Ruminococcus obeum ATCC 29174 Scfld023 genomic scaffold, whole genome shotgun sequence	2611
NZ_DS264291.1	Ruminococcus obeum ATCC 29174 Scfld022 genomic scaffold, whole genome shotgun sequence	2774
NZ_DS264290.1	Ruminococcus obeum ATCC 29174 Scfld021 genomic scaffold, whole genome shotgun sequence	2817
NZ_DS264289.1	Ruminococcus obeum ATCC 29174 Scfld020 genomic scaffold, whole genome shotgun sequence	178566
NZ_DS264383.1	Ruminococcus torques ATCC 27756 Scfld0240 genomic scaffold, whole genome shotgun sequence	99745
NZ_DS264382.1	Ruminococcus torques ATCC 27756 Scfld0239 genomic scaffold, whole genome shotgun sequence	88552
NZ_DS264381.1	Ruminococcus torques ATCC 27756 Scfld0238 genomic scaffold, whole genome shotgun sequence	700
NZ_DS264380.1	Ruminococcus torques ATCC 27756 Scfld0237 genomic scaffold, whole genome shotgun sequence	670
NZ_DS264379.1	Ruminococcus torques ATCC 27756 Scfld0236 genomic scaffold, whole genome shotgun sequence	115
NZ_DS264378.1	Ruminococcus torques ATCC 27756 Scfld0235 genomic scaffold, whole genome shotgun sequence	11745
NZ_DS264377.1	Ruminococcus torques ATCC 27756 Scfld0234 genomic scaffold, whole genome shotgun sequence	10147
NZ_DS264376.1	Ruminococcus torques ATCC 27756 Scfld0233 genomic scaffold, whole genome shotgun sequence	113538
NZ_DS264375.1	Ruminococcus torques ATCC 27756 Scfld0232 genomic scaffold, whole genome shotgun sequence	865

NZ_DS264374.1	Ruminococcus torques ATCC 27756 Scfld0231 genomic scaffold, whole genome shotgun sequence	51351
NZ_DS264373.1	Ruminococcus torques ATCC 27756 Scfld0230 genomic scaffold, whole genome shotgun sequence	117
NZ_DS264372.1	Ruminococcus torques ATCC 27756 Scfld0229 genomic scaffold, whole genome shotgun sequence	15093
NZ_DS264371.1	Ruminococcus torques ATCC 27756 Scfld0228 genomic scaffold, whole genome shotgun sequence	17937
NZ_DS264370.1	Ruminococcus torques ATCC 27756 Scfld0227 genomic scaffold, whole genome shotgun sequence	22216
NZ_DS264369.1	Ruminococcus torques ATCC 27756 Scfld0226 genomic scaffold, whole genome shotgun sequence	27026
NZ_DS264368.1	Ruminococcus torques ATCC 27756 Scfld0225 genomic scaffold, whole genome shotgun sequence	10424
NZ_DS264367.1	Ruminococcus torques ATCC 27756 Scfld0224 genomic scaffold, whole genome shotgun sequence	100621
NZ_DS264366.1	Ruminococcus torques ATCC 27756 Scfld0223 genomic scaffold, whole genome shotgun sequence	57511
NZ_DS264365.1	Ruminococcus torques ATCC 27756 Scfld0222 genomic scaffold, whole genome shotgun sequence	66704
NZ_DS264364.1	Ruminococcus torques ATCC 27756 Scfld0221 genomic scaffold, whole genome shotgun sequence	89085
NZ_DS264363.1	Ruminococcus torques ATCC 27756 Scfld0220 genomic scaffold, whole genome shotgun sequence	25388
NZ_DS264362.1	Ruminococcus torques ATCC 27756 Scfld0219 genomic scaffold, whole genome shotgun sequence	856
NZ_DS264361.1	Ruminococcus torques ATCC 27756 Scfld0218 genomic scaffold, whole genome shotgun sequence	807
NZ_DS264360.1	Ruminococcus torques ATCC 27756 Scfld0217 genomic scaffold, whole genome shotgun sequence	842
NZ_DS264359.1	Ruminococcus torques ATCC 27756 Scfld0216 genomic	94999

	scaffold, whole genome shotgun sequence	
NZ_DS264358.1	Ruminococcus torques ATCC 27756 Scfld0215 genomic scaffold, whole genome shotgun sequence	209206
NZ_DS264357.1	Ruminococcus torques ATCC 27756 Scfld0214 genomic scaffold, whole genome shotgun sequence	8182
NZ_DS264356.1	Ruminococcus torques ATCC 27756 Scfld0213 genomic scaffold, whole genome shotgun sequence	251026
NZ_DS264355.1	Ruminococcus torques ATCC 27756 Scfld0212 genomic scaffold, whole genome shotgun sequence	4802
NZ_DS264354.1	Ruminococcus torques ATCC 27756 Scfld0211 genomic scaffold, whole genome shotgun sequence	2483
NZ_DS264353.1	Ruminococcus torques ATCC 27756 Scfld0210 genomic scaffold, whole genome shotgun sequence	3504
NZ_DS264352.1	Ruminococcus torques ATCC 27756 Scfld029 genomic scaffold, whole genome shotgun sequence	9990
NZ_DS264351.1	Ruminococcus torques ATCC 27756 Scfld028 genomic scaffold, whole genome shotgun sequence	6036
NZ_DS264350.1	Ruminococcus torques ATCC 27756 Scfld027 genomic scaffold, whole genome shotgun sequence	6036
NZ_DS264349.1	Ruminococcus torques ATCC 27756 Scfld026 genomic scaffold, whole genome shotgun sequence	165245
NZ_DS264348.1	Ruminococcus torques ATCC 27756 Scfld025 genomic scaffold, whole genome shotgun sequence	4210
NZ_DS264347.1	Ruminococcus torques ATCC 27756 Scfld024 genomic scaffold, whole genome shotgun sequence	81754
NZ_DS264346.1	Ruminococcus torques ATCC 27756 Scfld023 genomic scaffold, whole genome shotgun sequence	125295
NZ_DS264345.1	Ruminococcus torques ATCC 27756 Scfld022 genomic scaffold, whole genome shotgun sequence	710081
NZ_DS264344.1	Ruminococcus torques ATCC 27756 Scfld021 genomic scaffold, whole genome shotgun sequence	223398

NZ_DS264343.1	Ruminococcus torques ATCC 27756 Scfld020 genomic scaffold, whole genome shotgun sequence	23404
NZ_DS264419.1	Dorea longicatena DSM 13814 Scfld0235 genomic scaffold, whole genome shotgun sequence	139199
NZ_DS264418.1	Dorea longicatena DSM 13814 Scfld0234 genomic scaffold, whole genome shotgun sequence	177295
NZ_DS264417.1	Dorea longicatena DSM 13814 Scfld0233 genomic scaffold, whole genome shotgun sequence	22544
NZ_DS264416.1	Dorea longicatena DSM 13814 Scfld0232 genomic scaffold, whole genome shotgun sequence	116100
NZ_DS264415.1	Dorea longicatena DSM 13814 Scfld0231 genomic scaffold, whole genome shotgun sequence	410383
NZ_DS264414.1	Dorea longicatena DSM 13814 Scfld0230 genomic scaffold, whole genome shotgun sequence	76683
NZ_DS264413.1	Dorea longicatena DSM 13814 Scfld0229 genomic scaffold, whole genome shotgun sequence	7496
NZ_DS264412.1	Dorea longicatena DSM 13814 Scfld0228 genomic scaffold, whole genome shotgun sequence	6578
NZ_DS264411.1	Dorea longicatena DSM 13814 Scfld0227 genomic scaffold, whole genome shotgun sequence	11821
NZ_DS264410.1	Dorea longicatena DSM 13814 Scfld0226 genomic scaffold, whole genome shotgun sequence	27850
NZ_DS264409.1	Dorea longicatena DSM 13814 Scfld0225 genomic scaffold, whole genome shotgun sequence	29809
NZ_DS264408.1	Dorea longicatena DSM 13814 Scfld0224 genomic scaffold, whole genome shotgun sequence	34468
NZ_DS264407.1	Dorea longicatena DSM 13814 Scfld0223 genomic scaffold, whole genome shotgun sequence	38061
NZ_DS264406.1	Dorea longicatena DSM 13814 Scfld0222 genomic scaffold, whole genome shotgun sequence	78319
NZ_DS264405.1	Dorea longicatena DSM 13814 Scfld0221 genomic	72941

	scaffold, whole genome shotgun sequence	
NZ_DS264404.1	Dorea longicatena DSM 13814 Scfld0220 genomic scaffold, whole genome shotgun sequence	49524
NZ_DS264403.1	Dorea longicatena DSM 13814 Scfld0219 genomic scaffold, whole genome shotgun sequence	45355
NZ_DS264402.1	Dorea longicatena DSM 13814 Scfld0218 genomic scaffold, whole genome shotgun sequence	76446
NZ_DS264401.1	Dorea longicatena DSM 13814 Scfld0217 genomic scaffold, whole genome shotgun sequence	92828
NZ_DS264400.1	Dorea longicatena DSM 13814 Scfld0216 genomic scaffold, whole genome shotgun sequence	22817
NZ_DS264399.1	Dorea longicatena DSM 13814 Scfld0215 genomic scaffold, whole genome shotgun sequence	81779
NZ_DS264398.1	Dorea longicatena DSM 13814 Scfld0214 genomic scaffold, whole genome shotgun sequence	90003
NZ_DS264397.1	Dorea longicatena DSM 13814 Scfld0213 genomic scaffold, whole genome shotgun sequence	4470
NZ_DS264396.1	Dorea longicatena DSM 13814 Scfld0212 genomic scaffold, whole genome shotgun sequence	1738
NZ_DS264395.1	Dorea longicatena DSM 13814 Scfld0211 genomic scaffold, whole genome shotgun sequence	3339
NZ_DS264394.1	Dorea longicatena DSM 13814 Scfld0210 genomic scaffold, whole genome shotgun sequence	5832
NZ_DS264393.1	Dorea longicatena DSM 13814 Scfld029 genomic scaffold, whole genome shotgun sequence	216476
NZ_DS264392.1	Dorea longicatena DSM 13814 Scfld028 genomic scaffold, whole genome shotgun sequence	539999
NZ_DS264391.1	Dorea longicatena DSM 13814 Scfld027 genomic scaffold, whole genome shotgun sequence	283525
NZ_DS264390.1	Dorea longicatena DSM 13814 Scfld026 genomic scaffold, whole genome shotgun sequence	3539

NZ_DS264389.1	Dorea longicatena DSM 13814 Scfld025 genomic scaffold, whole genome shotgun sequence	84037
NZ_DS264388.1	Dorea longicatena DSM 13814 Scfld024 genomic scaffold, whole genome shotgun sequence	5180
NZ_DS264387.1	Dorea longicatena DSM 13814 Scfld023 genomic scaffold, whole genome shotgun sequence	2540
NZ_DS264386.1	Dorea longicatena DSM 13814 Scfld022 genomic scaffold, whole genome shotgun sequence	2677
NZ_DS264385.1	Dorea longicatena DSM 13814 Scfld021 genomic scaffold, whole genome shotgun sequence	3220
NZ_DS264384.1	Dorea longicatena DSM 13814 Scfld020 genomic scaffold, whole genome shotgun sequence	50562
NZ_DS480351.1	Clostridium leptum DSM 753 Scfld_02_20 genomic scaffold, whole genome shotgun sequence	3450
NZ_DS480350.1	Clostridium leptum DSM 753 Scfld_02_19 genomic scaffold, whole genome shotgun sequence	523705
NZ_DS480349.1	Clostridium leptum DSM 753 Scfld_02_18 genomic scaffold, whole genome shotgun sequence	564969
NZ_DS480348.1	Clostridium leptum DSM 753 Scfld_02_17 genomic scaffold, whole genome shotgun sequence	498680
NZ_DS480347.1	Clostridium leptum DSM 753 Scfld_02_16 genomic scaffold, whole genome shotgun sequence	452649
NZ_DS480346.1	Clostridium leptum DSM 753 Scfld_02_15 genomic scaffold, whole genome shotgun sequence	222869
NZ_DS480345.1	Clostridium leptum DSM 753 Scfld_02_14 genomic scaffold, whole genome shotgun sequence	266505
NZ_DS480344.1	Clostridium leptum DSM 753 Scfld_02_13 genomic scaffold, whole genome shotgun sequence	99329
NZ_DS480343.1	Clostridium leptum DSM 753 Scfld_02_12 genomic scaffold, whole genome shotgun sequence	168688
NZ_DS480342.1	Clostridium leptum DSM 753 Scfld_02_11 genomic	99119

	scaffold, whole genome shotgun sequence	
NZ_DS480341.1	Clostridium leptum DSM 753 Scfld_02_10 genomic scaffold, whole genome shotgun sequence	90123
NZ_DS480340.1	Clostridium leptum DSM 753 Scfld_02_9 genomic scaffold, whole genome shotgun sequence	58025
NZ_DS480339.1	Clostridium leptum DSM 753 Scfld_02_8 genomic scaffold, whole genome shotgun sequence	27481
NZ_DS480338.1	Clostridium leptum DSM 753 Scfld_02_7 genomic scaffold, whole genome shotgun sequence	47797
NZ_DS480337.1	Clostridium leptum DSM 753 Scfld_02_6 genomic scaffold, whole genome shotgun sequence	12535
NZ_DS480336.1	Clostridium leptum DSM 753 Scfld_02_5 genomic scaffold, whole genome shotgun sequence	40142
NZ_DS480335.1	Clostridium leptum DSM 753 Scfld_02_4 genomic scaffold, whole genome shotgun sequence	34044
NZ_DS480334.1	Clostridium leptum DSM 753 Scfld_02_3 genomic scaffold, whole genome shotgun sequence	26694
NZ_DS480333.1	Clostridium leptum DSM 753 Scfld_02_2 genomic scaffold, whole genome shotgun sequence	19864
NZ_DS480332.1	Clostridium leptum DSM 753 Scfld_02_1 genomic scaffold, whole genome shotgun sequence	7141
NZ_DS480331.1	Clostridium leptum DSM 753 Scfld_02_0 genomic scaffold, whole genome shotgun sequence	6400
NZ_DS483503.1	Faecalibacterium prausnitzii M21/2 Scfld_02_24 genomic scaffold, whole genome shotgun sequence	999620
NZ_DS483502.1	Faecalibacterium prausnitzii M21/2 Scfld_02_23 genomic scaffold, whole genome shotgun sequence	207415
NZ_DS483501.1	Faecalibacterium prausnitzii M21/2 Scfld_02_22 genomic scaffold, whole genome shotgun sequence	153037
NZ_DS483500.1	Faecalibacterium prausnitzii M21/2 Scfld_02_21 genomic scaffold, whole genome shotgun sequence	296445

NZ_DS483499.1	Faecalibacterium prausnitzii M21/2 Scfld_02_20 genomic scaffold, whole genome shotgun sequence	155107
NZ_DS483498.1	Faecalibacterium prausnitzii M21/2 Scfld_02_19 genomic scaffold, whole genome shotgun sequence	96475
NZ_DS483497.1	Faecalibacterium prausnitzii M21/2 Scfld_02_18 genomic scaffold, whole genome shotgun sequence	103074
NZ_DS483496.1	Faecalibacterium prausnitzii M21/2 Scfld_02_17 genomic scaffold, whole genome shotgun sequence	88016
NZ_DS483495.1	Faecalibacterium prausnitzii M21/2 Scfld_02_16 genomic scaffold, whole genome shotgun sequence	102896
NZ_DS483494.1	Faecalibacterium prausnitzii M21/2 Scfld_02_15 genomic scaffold, whole genome shotgun sequence	105972
NZ_DS483493.1	Faecalibacterium prausnitzii M21/2 Scfld_02_14 genomic scaffold, whole genome shotgun sequence	71339
NZ_DS483492.1	Faecalibacterium prausnitzii M21/2 Scfld_02_13 genomic scaffold, whole genome shotgun sequence	47391
NZ_DS483491.1	Faecalibacterium prausnitzii M21/2 Scfld_02_12 genomic scaffold, whole genome shotgun sequence	50695
NZ_DS483490.1	Faecalibacterium prausnitzii M21/2 Scfld_02_11 genomic scaffold, whole genome shotgun sequence	42964
NZ_DS483489.1	Faecalibacterium prausnitzii M21/2 Scfld_02_10 genomic scaffold, whole genome shotgun sequence	30118
NZ_DS483488.1	Faecalibacterium prausnitzii M21/2 Scfld_02_9 genomic scaffold, whole genome shotgun sequence	20250
NZ_DS483487.1	Faecalibacterium prausnitzii M21/2 Scfld_02_8 genomic scaffold, whole genome shotgun sequence	27529
NZ_DS483486.1	Faecalibacterium prausnitzii M21/2 Scfld_02_7 genomic scaffold, whole genome shotgun sequence	13467
NZ_DS483485.1	Faecalibacterium prausnitzii M21/2 Scfld_02_6 genomic scaffold, whole genome shotgun sequence	19145
NZ_DS483484.1	Faecalibacterium prausnitzii M21/2 Scfld_02_5 genomic	7326

	scaffold, whole genome shotgun sequence	
NZ_DS483483.1	Faecalibacterium prausnitzii M21/2 Scfld_02_4 genomic scaffold, whole genome shotgun sequence	9202
NZ_DS483482.1	Faecalibacterium prausnitzii M21/2 Scfld_02_3 genomic scaffold, whole genome shotgun sequence	407782
NZ_DS483481.1	Faecalibacterium prausnitzii M21/2 Scfld_02_2 genomic scaffold, whole genome shotgun sequence	2037
NZ_DS483480.1	Faecalibacterium prausnitzii M21/2 Scfld_02_1 genomic scaffold, whole genome shotgun sequence	68459
NZ_DS483479.1	Faecalibacterium prausnitzii M21/2 Scfld_02_0 genomic scaffold, whole genome shotgun sequence	1622
NZ_DS483542.1	Coprococcus eutactus ATCC 27759 Scfld_02_22 genomic scaffold, whole genome shotgun sequence	195117
NZ_DS483541.1	Coprococcus eutactus ATCC 27759 Scfld_02_21 genomic scaffold, whole genome shotgun sequence	2590
NZ_DS483540.1	Coprococcus eutactus ATCC 27759 Scfld_02_20 genomic scaffold, whole genome shotgun sequence	92864
NZ_DS483539.1	Coprococcus eutactus ATCC 27759 Scfld_02_19 genomic scaffold, whole genome shotgun sequence	77672
NZ_DS483538.1	Coprococcus eutactus ATCC 27759 Scfld_02_18 genomic scaffold, whole genome shotgun sequence	92796
NZ_DS483537.1	Coprococcus eutactus ATCC 27759 Scfld_02_17 genomic scaffold, whole genome shotgun sequence	110911
NZ_DS483536.1	Coprococcus eutactus ATCC 27759 Scfld_02_16 genomic scaffold, whole genome shotgun sequence	105596
NZ_DS483535.1	Coprococcus eutactus ATCC 27759 Scfld_02_15 genomic scaffold, whole genome shotgun sequence	2324
NZ_DS483534.1	Coprococcus eutactus ATCC 27759 Scfld_02_14 genomic scaffold, whole genome shotgun sequence	172583
NZ_DS483533.1	Coprococcus eutactus ATCC 27759 Scfld_02_13 genomic scaffold, whole genome shotgun sequence	118189

NZ_DS483532.1	Coprococcus eutactus ATCC 27759 Scfld_02_12 genomic scaffold, whole genome shotgun sequence	624153
NZ_DS483531.1	Coprococcus eutactus ATCC 27759 Scfld_02_11 genomic scaffold, whole genome shotgun sequence	6057
NZ_DS483530.1	Coprococcus eutactus ATCC 27759 Scfld_02_10 genomic scaffold, whole genome shotgun sequence	3723
NZ_DS483529.1	Coprococcus eutactus ATCC 27759 Scfld_02_9 genomic scaffold, whole genome shotgun sequence	215819
NZ_DS483528.1	Coprococcus eutactus ATCC 27759 Scfld_02_8 genomic scaffold, whole genome shotgun sequence	60644
NZ_DS483527.1	Coprococcus eutactus ATCC 27759 Scfld_02_7 genomic scaffold, whole genome shotgun sequence	128678
NZ_DS483526.1	Coprococcus eutactus ATCC 27759 Scfld_02_6 genomic scaffold, whole genome shotgun sequence	371583
NZ_DS483525.1	Coprococcus eutactus ATCC 27759 Scfld_02_5 genomic scaffold, whole genome shotgun sequence	180987
NZ_DS483524.1	Coprococcus eutactus ATCC 27759 Scfld_02_4 genomic scaffold, whole genome shotgun sequence	1619
NZ_DS483523.1	Coprococcus eutactus ATCC 27759 Scfld_02_3 genomic scaffold, whole genome shotgun sequence	254280
NZ_DS483522.1	Coprococcus eutactus ATCC 27759 Scfld_02_2 genomic scaffold, whole genome shotgun sequence	3252
NZ_DS483521.1	Coprococcus eutactus ATCC 27759 Scfld_02_1 genomic scaffold, whole genome shotgun sequence	2931
NZ_DS483520.1	Coprococcus eutactus ATCC 27759 Scfld_02_0 genomic scaffold, whole genome shotgun sequence	278619
NZ_DS499581.1	Alistipes putredinis DSM 17216 Scfld_02_11 genomic scaffold, whole genome shotgun sequence	227614
NZ_DS499580.1	Alistipes putredinis DSM 17216 Scfld_02_10 genomic scaffold, whole genome shotgun sequence	479642
NZ_DS499579.1	Alistipes putredinis DSM 17216 Scfld_02_9 genomic	626692

	scaffold, whole genome shotgun sequence	
NZ_DS499578.1	Alistipes putredinis DSM 17216 Scfld_02_8 genomic scaffold, whole genome shotgun sequence	3853
NZ_DS499577.1	Alistipes putredinis DSM 17216 Scfld_02_7 genomic scaffold, whole genome shotgun sequence	1197641
NZ_DS499576.1	Alistipes putredinis DSM 17216 Scfld_02_6 genomic scaffold, whole genome shotgun sequence	3644
NZ_DS499575.1	Alistipes putredinis DSM 17216 Scfld_02_5 genomic scaffold, whole genome shotgun sequence	1519
NZ_DS499574.1	Alistipes putredinis DSM 17216 Scfld_02_4 genomic scaffold, whole genome shotgun sequence	1951
NZ_DS499573.1	Alistipes putredinis DSM 17216 Scfld_02_3 genomic scaffold, whole genome shotgun sequence	2558
NZ_DS499572.1	Alistipes putredinis DSM 17216 Scfld_02_2 genomic scaffold, whole genome shotgun sequence	2776
NZ_DS499571.1	Alistipes putredinis DSM 17216 Scfld_02_1 genomic scaffold, whole genome shotgun sequence	1039
NZ_DS499570.1	Alistipes putredinis DSM 17216 Scfld_02_0 genomic scaffold, whole genome shotgun sequence	1749
NZ_DS499677.1	Bacteroides stercoris ATCC 43183 Scfld_02_16 genomic scaffold, whole genome shotgun sequence	468184
NZ_DS499676.1	Bacteroides stercoris ATCC 43183 Scfld_02_15 genomic scaffold, whole genome shotgun sequence	722113
NZ_DS499675.1	Bacteroides stercoris ATCC 43183 Scfld_02_14 genomic scaffold, whole genome shotgun sequence	85502
NZ_DS499674.1	Bacteroides stercoris ATCC 43183 Scfld_02_13 genomic scaffold, whole genome shotgun sequence	695517
NZ_DS499673.1	Bacteroides stercoris ATCC 43183 Scfld_02_12 genomic scaffold, whole genome shotgun sequence	587129
NZ_DS499672.1	Bacteroides stercoris ATCC 43183 Scfld_02_11 genomic scaffold, whole genome shotgun sequence	476026

NZ_DS499671.1	Bacteroides stercoris ATCC 43183 Scfld_02_10 genomic scaffold, whole genome shotgun sequence	250100
NZ_DS499670.1	Bacteroides stercoris ATCC 43183 Scfld_02_9 genomic scaffold, whole genome shotgun sequence	65349
NZ_DS499669.1	Bacteroides stercoris ATCC 43183 Scfld_02_8 genomic scaffold, whole genome shotgun sequence	78874
NZ_DS499668.1	Bacteroides stercoris ATCC 43183 Scfld_02_7 genomic scaffold, whole genome shotgun sequence	72902
NZ_DS499667.1	Bacteroides stercoris ATCC 43183 Scfld_02_6 genomic scaffold, whole genome shotgun sequence	46117
NZ_DS499666.1	Bacteroides stercoris ATCC 43183 Scfld_02_5 genomic scaffold, whole genome shotgun sequence	66283
NZ_DS499665.1	Bacteroides stercoris ATCC 43183 Scfld_02_4 genomic scaffold, whole genome shotgun sequence	71251
NZ_DS499664.1	Bacteroides stercoris ATCC 43183 Scfld_02_3 genomic scaffold, whole genome shotgun sequence	24919
NZ_DS499663.1	Bacteroides stercoris ATCC 43183 Scfld_02_2 genomic scaffold, whole genome shotgun sequence	4587
NZ_DS499662.1	Bacteroides stercoris ATCC 43183 Scfld_02_1 genomic scaffold, whole genome shotgun sequence	281216
NZ_DS499661.1	Bacteroides stercoris ATCC 43183 Scfld_02_0 genomic scaffold, whole genome shotgun sequence	13760
NZ_AAVN02000022.1	Collinsella aerofaciens ATCC 25986 C_aerofaciens-2.0_Cont809, whole genome shotgun sequence	2111
NZ_AAVN02000021.1	Collinsella aerofaciens ATCC 25986 C_aerofaciens-2.0_Cont1.6, whole genome shotgun sequence	2932
NZ_AAVN02000020.1	Collinsella aerofaciens ATCC 25986 C_aerofaciens-2.0_Cont26.1, whole genome shotgun sequence	2949

NZ_AAVN02000019.1	Collinsella aerofaciens ATCC 25986 C_aerofaciens-2.0_Cont22.2, whole genome shotgun sequence	3244
NZ_AAVN02000018.1	Collinsella aerofaciens ATCC 25986 C_aerofaciens-2.0_Cont22.1, whole genome shotgun sequence	4270
NZ_AAVN02000017.1	Collinsella aerofaciens ATCC 25986 C_aerofaciens-2.0_Cont933, whole genome shotgun sequence	22286
NZ_AAVN02000016.1	Collinsella aerofaciens ATCC 25986 C_aerofaciens-2.0_Cont742, whole genome shotgun sequence	29625
NZ_AAVN02000015.1	Collinsella aerofaciens ATCC 25986 C_aerofaciens-2.0_Cont997, whole genome shotgun sequence	32993
NZ_AAVN02000014.1	Collinsella aerofaciens ATCC 25986 C_aerofaciens-2.0_Cont941, whole genome shotgun sequence	35550
NZ_AAVN02000013.1	Collinsella aerofaciens ATCC 25986 C_aerofaciens-2.0_Cont1002, whole genome shotgun sequence	44709
NZ_AAVN02000012.1	Collinsella aerofaciens ATCC 25986 C_aerofaciens-2.0_Cont982, whole genome shotgun sequence	49431
NZ_AAVN02000011.1	Collinsella aerofaciens ATCC 25986 C_aerofaciens-2.0_Cont1050, whole genome shotgun sequence	74115
NZ_AAVN02000010.1	Collinsella aerofaciens ATCC 25986 C_aerofaciens-2.0_Cont1049, whole genome shotgun sequence	77635
NZ_AAVN02000009.1	Collinsella aerofaciens ATCC 25986 C_aerofaciens-2.0_Cont994, whole genome shotgun sequence	105018
NZ_AAVN02000	Collinsella aerofaciens ATCC 25986	121015

008.1	C_aerofaciens-2.0_Cont911, whole genome shotgun sequence	
NZ_AAVN02000 007.1	Collinsella aerofaciens ATCC 25986 C_aerofaciens-2.0_Cont985, whole genome shotgun sequence	145096
NZ_AAVN02000 006.1	Collinsella aerofaciens ATCC 25986 C_aerofaciens-2.0_Cont931, whole genome shotgun sequence	169609
NZ_AAVN02000 005.1	Collinsella aerofaciens ATCC 25986 C_aerofaciens-2.0_Cont930, whole genome shotgun sequence	183388
NZ_AAVN02000 004.1	Collinsella aerofaciens ATCC 25986 C_aerofaciens-2.0_Cont973, whole genome shotgun sequence	184100
NZ_AAVN02000 003.1	Collinsella aerofaciens ATCC 25986 C_aerofaciens-2.0_Cont981, whole genome shotgun sequence	280878
NZ_AAVN02000 002.1	Collinsella aerofaciens ATCC 25986 C_aerofaciens-2.0_Cont899, whole genome shotgun sequence	386275
NZ_AAVN02000 001.1	Collinsella aerofaciens ATCC 25986 C_aerofaciens-2.0_Cont1020, whole genome shotgun sequence	478714
NZ_AAVN02000 025.1	Collinsella aerofaciens ATCC 25986 C_aerofaciens-2.0_Cont366, whole genome shotgun sequence	1667
NZ_AAVN02000 024.1	Collinsella aerofaciens ATCC 25986 C_aerofaciens-2.0_Cont30.1, whole genome shotgun sequence	1185
NZ_AAVN02000 023.1	Collinsella aerofaciens ATCC 25986 C_aerofaciens-2.0_Cont18.1, whole genome shotgun sequence	1074
NZ_JH724260.1	Bacteroides uniformis CL03T00C23 genomic scaffold supercont1.1, whole genome shotgun sequence	4915651

NZ_JH724261.1	Bacteroides uniformis CL03T00C23 genomic scaffold supercont1.2, whole genome shotgun sequence	20182
NZ_JH724262.1	Bacteroides uniformis CL03T00C23 genomic scaffold supercont1.3, whole genome shotgun sequence	11247
NZ_JH724263.1	Bacteroides uniformis CL03T00C23 genomic scaffold supercont1.4, whole genome shotgun sequence	4778
NZ_JH724264.1	Bacteroides uniformis CL03T00C23 genomic scaffold supercont1.5, whole genome shotgun sequence	2930
NZ_JH724265.1	Bacteroides uniformis CL03T00C23 genomic scaffold supercont1.6, whole genome shotgun sequence	1557
NZ_JH724266.1	Bacteroides uniformis CL03T00C23 genomic scaffold supercont1.7, whole genome shotgun sequence	1421
NZ_JH724267.1	Bacteroides uniformis CL03T00C23 genomic scaffold supercont1.8, whole genome shotgun sequence	1175
NZ_JH976524.1	Parabacteroides merdae CL09T00C40 genomic scaffold supercont1.1, whole genome shotgun sequence	928763
NZ_JH976525.1	Parabacteroides merdae CL09T00C40 genomic scaffold supercont1.2, whole genome shotgun sequence	901995
NZ_JH976526.1	Parabacteroides merdae CL09T00C40 genomic scaffold supercont1.3, whole genome shotgun sequence	734811
NZ_JH976527.1	Parabacteroides merdae CL09T00C40 genomic scaffold supercont1.4, whole genome shotgun sequence	623804
NZ_JH976528.1	Parabacteroides merdae CL09T00C40 genomic scaffold supercont1.5, whole genome shotgun sequence	454576
NZ_JH976529.1	Parabacteroides merdae CL09T00C40 genomic scaffold supercont1.6, whole genome shotgun sequence	430807
NZ_JH976530.1	Parabacteroides merdae CL09T00C40 genomic scaffold supercont1.7, whole genome shotgun sequence	175002
NZ_JH976531.1	Parabacteroides merdae CL09T00C40 genomic scaffold supercont1.8, whole genome shotgun sequence	132113
NZ_JH976532.1	Parabacteroides merdae CL09T00C40 genomic scaffold	53605

	supercont1.9, whole genome shotgun sequence	
NZ_JH976533.1	Parabacteroides merdae CL09T00C40 genomic scaffold supercont1.10, whole genome shotgun sequence	20053
NZ_JH976534.1	Parabacteroides merdae CL09T00C40 genomic scaffold supercont1.11, whole genome shotgun sequence	3212
NZ_KB907512.1	Eubacterium siraeum DSM 15702 genomic scaffold G397DRAFT_scaffold00001.1, whole genome shotgun sequence	365367
NZ_KB907513.1	Eubacterium siraeum DSM 15702 genomic scaffold G397DRAFT_scaffold00002.2, whole genome shotgun sequence	272735
NZ_KB907514.1	Eubacterium siraeum DSM 15702 genomic scaffold G397DRAFT_scaffold00003.3, whole genome shotgun sequence	211437
NZ_KB907515.1	Eubacterium siraeum DSM 15702 genomic scaffold G397DRAFT_scaffold00004.4, whole genome shotgun sequence	204201
NZ_KB907516.1	Eubacterium siraeum DSM 15702 genomic scaffold G397DRAFT_scaffold00005.5, whole genome shotgun sequence	144697
NZ_KB907517.1	Eubacterium siraeum DSM 15702 genomic scaffold G397DRAFT_scaffold00006.6, whole genome shotgun sequence	139720
NZ_KB907518.1	Eubacterium siraeum DSM 15702 genomic scaffold G397DRAFT_scaffold00007.7, whole genome shotgun sequence	130756
NZ_KB907519.1	Eubacterium siraeum DSM 15702 genomic scaffold G397DRAFT_scaffold00008.8, whole genome shotgun sequence	123324
NZ_KB907520.1	Eubacterium siraeum DSM 15702 genomic scaffold G397DRAFT_scaffold00009.9, whole genome shotgun sequence	107991
NZ_KB907521.1	Eubacterium siraeum DSM 15702 genomic scaffold	103320

	G397DRAFT_scaffold00010.10, whole genome shotgun sequence	
NZ_KB907522.1	Eubacterium siraeum DSM 15702 genomic scaffold G397DRAFT_scaffold00011.11, whole genome shotgun sequence	100435
NZ_KB907523.1	Eubacterium siraeum DSM 15702 genomic scaffold G397DRAFT_scaffold00012.12, whole genome shotgun sequence	100048
NZ_KB907524.1	Eubacterium siraeum DSM 15702 genomic scaffold G397DRAFT_scaffold00013.13, whole genome shotgun sequence	81093
NZ_KB907525.1	Eubacterium siraeum DSM 15702 genomic scaffold G397DRAFT_scaffold00014.14, whole genome shotgun sequence	78078
NZ_KB907526.1	Eubacterium siraeum DSM 15702 genomic scaffold G397DRAFT_scaffold00015.15, whole genome shotgun sequence	71742
NZ_KB907527.1	Eubacterium siraeum DSM 15702 genomic scaffold G397DRAFT_scaffold00016.16, whole genome shotgun sequence	62876
NZ_KB907528.1	Eubacterium siraeum DSM 15702 genomic scaffold G397DRAFT_scaffold00017.17, whole genome shotgun sequence	57551
NZ_KB907529.1	Eubacterium siraeum DSM 15702 genomic scaffold G397DRAFT_scaffold00018.18, whole genome shotgun sequence	55033
NZ_KB907530.1	Eubacterium siraeum DSM 15702 genomic scaffold G397DRAFT_scaffold00019.19, whole genome shotgun sequence	42314
NZ_KB907531.1	Eubacterium siraeum DSM 15702 genomic scaffold G397DRAFT_scaffold00020.20, whole genome shotgun sequence	38612
NZ_KB907532.1	Eubacterium siraeum DSM 15702 genomic scaffold G397DRAFT_scaffold00021.21, whole genome shotgun	34396

	sequence	
NZ_KB907533.1	Eubacterium siraeum DSM 15702 genomic scaffold G397DRAFT_scaffold00022.22, whole genome shotgun sequence	24291
NZ_KB907534.1	Eubacterium siraeum DSM 15702 genomic scaffold G397DRAFT_scaffold00023.23, whole genome shotgun sequence	23012
NZ_KB907535.1	Eubacterium siraeum DSM 15702 genomic scaffold G397DRAFT_scaffold00024.24, whole genome shotgun sequence	19197
NZ_KB907536.1	Eubacterium siraeum DSM 15702 genomic scaffold G397DRAFT_scaffold00025.25, whole genome shotgun sequence	17524
NZ_KB907537.1	Eubacterium siraeum DSM 15702 genomic scaffold G397DRAFT_scaffold00026.26, whole genome shotgun sequence	14677
NZ_KB907538.1	Eubacterium siraeum DSM 15702 genomic scaffold G397DRAFT_scaffold00027.27, whole genome shotgun sequence	12203
NZ_KB907539.1	Eubacterium siraeum DSM 15702 genomic scaffold G397DRAFT_scaffold00028.28, whole genome shotgun sequence	6690
NZ_KB907540.1	Eubacterium siraeum DSM 15702 genomic scaffold G397DRAFT_scaffold00029.29, whole genome shotgun sequence	7290
NZ_KB907541.1	Eubacterium siraeum DSM 15702 genomic scaffold G397DRAFT_scaffold00030.30, whole genome shotgun sequence	5061
NZ_KB907542.1	Eubacterium siraeum DSM 15702 genomic scaffold G397DRAFT_scaffold00031.31, whole genome shotgun sequence	3305
NZ_KB907543.1	Eubacterium siraeum DSM 15702 genomic scaffold G397DRAFT_scaffold00032.32, whole genome shotgun sequence	2159

NZ_KB907544.1	Eubacterium siraeum DSM 15702 genomic scaffold G397DRAFT_scaffold00033.33, whole genome shotgun sequence	1694
NZ_KB907545.1	Eubacterium siraeum DSM 15702 genomic scaffold G397DRAFT_scaffold00034.34, whole genome shotgun sequence	1206
NZ_JAGQ01000 001.1	Ruminococcus gnavus AGR2154 N769DRAFT_scf7180000000026_quiver.1_C, whole genome shotgun sequence	14340
NZ_JAGQ01000 002.1	Ruminococcus gnavus AGR2154 N769DRAFT_scf7180000000027_quiver.2_C, whole genome shotgun sequence	1425222
NZ_JAGQ01000 003.1	Ruminococcus gnavus AGR2154 N769DRAFT_scf7180000000025_quiver.3_C, whole genome shotgun sequence	9222
NZ_JAGQ01000 004.1	Ruminococcus gnavus AGR2154 N769DRAFT_scf7180000000028_quiver.4_C, whole genome shotgun sequence	2261011
NZ_JAGQ01000 005.1	Ruminococcus gnavus AGR2154 N769DRAFT_scf7180000000024_quiver.5_C, whole genome shotgun sequence	14282
NZ_CP012938.1	Bacteroides ovatus strain ATCC 8483, complete genome	6472489

Table 21: Complete record of sequences with accession number used in the experiment of the lean and obese metagenomes.

References in Table 2

1. Meyer, Folker, et al. "The metagenomics RAST server–a public resource for the automatic phylogenetic and functional analysis of metagenomes." BMC bioinformatics 9.1 (2008): 1.
2. Huson DH, Auch AF, Qi J, Schuster SC. MEGAN analysis of metagenomic data. Genome Res. 2007;17(3):377–86.
3. Huson et al, Integrative analysis of environmental sequences using MEGAN4, Genome Res, 2011, 21:1552-1560.
4. Hunter, Sarah, et al. "EBI metagenomics—a new resource for the analysis and archiving of metagenomic data." Nucleic acids research 42.D1 (2014): D600-D606.
5. Kultima, Jens Roat, et al. "MOCAT: a metagenomics assembly and gene prediction toolkit." *PloS one* 7.10 (2012): e47656.
6. Su, Xiaoquan, Jian Xu, and Kang Ning. "Parallel-META: efficient metagenomic data analysis based on high-performance computation." *BMC Systems Biology* 6.1 (2012): 1.
7. Robertsen, Espen Mikal, et al. "META-pipe-Pipeline Annotation, Analysis and Visualization of Marine Metagenomic Sequence Data." *arXiv preprint arXiv:1604.04103* (2016).
8. Gerlach, Wolfgang, et al. "WebCARMA: a web application for the functional and taxonomic classification of unassembled metagenomic reads." *Bmc Bioinformatics* 10.1 (2009): 1.
9. Brady, A., & Salzberg, S. L. (2009). Phymm and PhymmBL: metagenomic phylogenetic classification with interpolated Markov models. Nature methods,6(9), 673-676.
10. Bohnebeck, U., Lombardot, T., Kottmann, R., & Glöckner, F. O. (2008). MetaMine–a tool to detect and analyse gene patterns in their environmental context. BMC bioinformatics, 9(1), 1.
